# Bipolar Palladium Membrane Enabling Crossover‐Free Selective Proton Transport

**DOI:** 10.1002/advs.76415

**Published:** 2026-07-06

**Authors:** Jiyeon Baek, Yeongbae Jeon, Seunga Lee, Ahee Choi, Joonmok Shim, Kyungho Lee, Ho Woun Jung, Sun Hyung Kim, Churl Hee Cho, Hyung Chul Yoon, Yun Jeong Hwang, Jae Hyung Kim

**Affiliations:** ^1^ Clean Fuel Research Laboratory Korea Institute of Energy Research Daejeon Republic of Korea; ^2^ Graduate School of Energy Science and Technology Chungnam National University Daejeon Republic of Korea; ^3^ Department of Chemistry College of Natural Science Seoul National University (SNU) Seoul Republic of Korea

**Keywords:** electrochemistry, ion transporter, materials science, membrane, proton transport

## Abstract

Establishing a proton‐selective membrane, capable of completely blocking the crossover of other chemical species, has been regarded as a long‐sought‐after goal for the stable and efficient operation of electrochemical devices. Conventional polymeric membranes suffer from an inherent trade‐off between conductivity and selectivity, imposed by their water‐mediated proton‐conduction mechanism, which precludes crossover‐free proton transport. Herein, we demonstrate a unique proton shuttling mechanism of the bipolar palladium membrane that enables selective proton transport while suppressing molecular crossover. Through bipolar electrochemical proton absorption and desorption, coupled with the lattice‐channeled hydrogen diffusion in palladium, the conductivity–selectivity relationship can be fundamentally circumvented. Using the bipolar electrochemical palladium membrane, we construct a water‐fed Li‐mediated N_2_ reduction system, suppressing water crossover between an aqueous anolyte and a non‐aqueous catholyte even under continuous flow and enabling water to serve as a sustainable proton source. We achieve an ammonia Faradaic efficiency of 51% and stable operation for 12 h under potential cycling. This study introduces a new ion transport mechanism, expanding the design space for next‐generation electrochemical systems that require strict compartmentalization.

## Graphical abstract

This study introduces a crossover‐free bipolar palladium membrane that transports protons selectively via reversible Pd/PdH*_x_* absorption–desorption and lattice hydrogen diffusion, overcoming the conductivity–selectivity trade‐off of polymer membranes. It enables water‐fed Li‐mediated ammonia synthesis, achieving 51% Faradaic efficiency and stable 12 h continuous‐flow operation without water crossover.

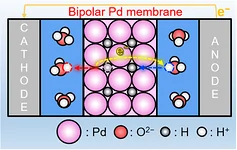

## 1 Introduction

Selective proton conduction while suppressing undesirable crossover of chemical species such as reactants, products, ions, or solvents between the cathode and anode is essential to secure the stable and efficient operation of electrochemical devices [1, 2, 3, 4, 5, 6]. In proton‐exchange‐membrane water electrolyzers, H_2_ crossover not only lowers coulombic efficiency but also increases H_2_ content in the O_2_ stream, creating explosive mixtures that force system shutdowns and reduce overall efficiency [5, 6]. Similar challenges arise in redox‐flow batteries, where unintended intermixing of redox‐active species across the ion‐exchange membrane (IEM) results in capacity fade and degradation of active materials, imposing the need for frequent electrolyte rebalancing or replacement [2, 7].

Such crossover‐induced limitations are even more severe in Li‐mediated electrochemical NH_3_ synthesis, which has recently been intensively studied as a sustainable NH_3_ production process [8, 9, 10]. To utilize H_2_O as a proton source, a hybrid electrolyte configuration has been established in which the Li‐mediated N_2_ reduction reaction (Li‐NRR) proceeds in an organic‐solvent‐based catholyte and the O_2_ evolution reaction (OER) is driven in an aqueous anolyte [11, 12, 13, 14]. These two electrolytes must be strictly separated, as the efficiency of Li‐NRR is highly sensitive to even trace amounts of water in the cathode and to unwanted oxidation reactions at the anode [15, 16, 17]. However, using conventional polymeric IEMs, H_2_O molecules inevitably permeate from the anolyte to the catholyte during the proton conduction. The H_2_O crossover leads to the formation of an unfavorable solid–electrolyte interphase (SEI) that passivates the cathode surface and limits the long‐term operation of Li‐NRR [11, 17].

To overcome these system‐level failures caused by the crossover, a broad spectrum of membrane engineering has been investigated, including modifications of ionomer [18] or multilayer design [19]. Nevertheless, completely preventing molecular crossover has remained elusive, mainly because polymeric porous membranes rely on water‐mediated ion transport. Ion conduction in the IEM stems from a combination of the vehicle mechanism, where hydrated ions migrate with their solvation shells, and the Grotthuss mechanism, where protons hop across hydrogen‐bonded water networks [20]. These pathways enable rapid proton transport but, in parallel, provide conduits for neutral species and gases, imposing an intrinsic trade‐off between conductivity and selectivity (Figure 1a) [21, 22, 23]. As a result, realizing proton conduction that is simultaneously efficient and free of crossover has remained a long‐standing and unresolved challenge in membrane science.

**FIGURE 1:**
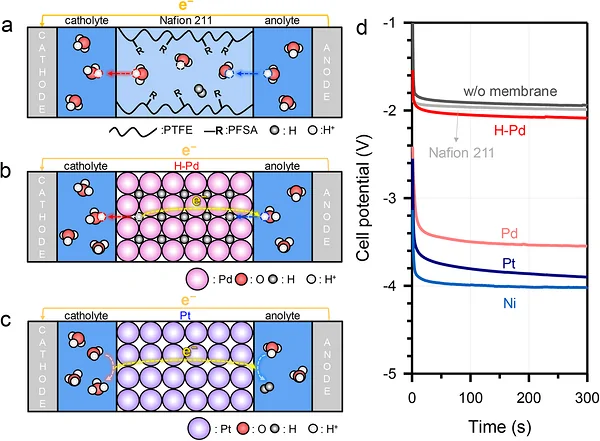
Proton transport mechanism of polymeric membrane and metal foils. Schematic illustrations of charge carrier transportation through (a) Nafion 211, (b) H‐Pd, and (c) Pt. PTFE and PFSA stand for polytetrafluoroethylene and perfluorosulfonic acid, respectively. (d) Cell potentials measured using different types of membranes in 0.1 M HClO_4_.

These limitations have motivated the exploration of alternative mechanisms for proton transport. Dense metal foils, intrinsically impermeable to molecular species, offer a promising direction as separators. However, when metal foils are used as the electrochemical membrane, they conduct electrons instead of protons, resulting in bipolar electrochemical oxidation and reduction reactions on each side of the foil [24]. This behavior of metal foil led us to hypothesize that if the bipolar electrochemical reduction and oxidation were driven by proton absorption (Pd + *x*H^+^ + *x*e^−^ → PdH*_x_*) and desorption (PdH*_x_* → Pd + *x*H^+^ + *x*e^−^) on Pd, respectively, coupled with the diffusion of hydrogen atoms within the Pd lattice, net proton transfer could be realized (Figure 1b). The fast kinetics for proton absorption and desorption on Pd [25] and the high hydrogen diffusivity in PdH*_x_* (*D* ≈ 1.3 × 10^−7^ cm^2^ s^−1^) [26] further support the viability of this mechanism. Consistent with this concept, Ye et al., recently reported an electrically isolated Pd membrane to transfer protons generated by water oxidation into a non‐aqueous Li‐NRR catholyte [14]. The selective proton transfer was verified across the Pd membrane by isotopic labeling experiments combined with online mass spectrometry, and they achieved an NH_3_ Faradaic efficiency (FE) of 36% at −6 mA cm^−2^ over 6 h.

In this work, we establish the operating mechanism of the bipolar electrochemical metal membrane by demonstrating the thermodynamic and kinetic competition between the proton absorption/desorption and electrolyte decomposition reactions. Spatially resolved potential profiling together with the quantitative analysis of competing reactions identifies the internal hydrogen content of Pd as the key determinant of proton transport efficiency, and Pt and Ni, lacking hydrogen storage capacity, cannot transfer protons. This electrochemical PdH*_x_*‐mediated proton shuttling mechanism allows a non‐aqueous Li‐NRR catholyte to be paired with an aqueous OER anolyte, enabling water to supply protons without water crossover. The system achieves an NH_3_ FE of 51% in a continuous‐flow gas‐N_2_‐fed electrolyzer and demonstrates stable operation for 12 h under potential cycling with minimal water crossover.

## 2 Results and Discussion

Given Pd's unique ability of electrochemical proton absorption and desorption [27], together with a high electronic conductivity of PdH (∼5 × 10^6^ S/m) [28], we posited that Pd could sustain a bipolar electrochemical proton‐pumping process that enables selective proton transport at room temperature. To examine this hypothesis, we constructed an H‐type electrolytic cell with a fixed inter‐electrode distance and evaluated cell potentials during water electrolysis in 0.1 M HClO_4_ by varying the membrane inserted between the electrodes (Figure S1a). In this configuration, two Pt plates were used as the cathode and anode, respectively.

Prior to using Pd as a membrane, we electrochemically preloaded hydrogen into Pd to prepare H‐Pd for facile hydrogen diffusion (Figures S1b and S2) [29]. X‐ray diffraction analysis confirmed the formation of the β‐PdH phase, verifying that hydrogen had been inserted into the Pd lattice (Figure S3). When H‐Pd was employed as the membrane under an applied current density of −1 mA cm^−2^, the resulting cell potential was −2.09 V (Figure 1d and Table S1), comparable to the cell potential obtained both without a membrane (w/o membrane, −1.97 V) and with conventional proton exchange membrane (Nafion 211, −1.99 V). The modest increase in cell potential in H‐Pd can be attributed to the overpotential associated with proton absorption and desorption (Pd + *x*H^+^ + *x*e^−^ ⇌ PdH*_x_*) at each side of the H‐Pd surfaces. To further validate the mechanistic origin, we replaced the H‐Pd membrane with other metals incapable of hydrogen storage. When Pt was inserted, the cell potential rose to almost twice (−3.9 V) that observed for H‐Pd. Because proton absorption or desorption is not favorable with Pt, H_2_ evolution reaction (HER) and OER instead occur separately at each side of the bipolar Pt foil (Figure 1c) [30]. As a result, the cell became equivalent to stacking two independent electrochemical cells, yielding approximately a twofold increase in potential without proton transport. The Ni membrane displayed a higher cell potential (−4 V) than Pt, originating from its inability to store hydrogen and lower catalytic activity for water electrolysis compared to Pt [31]. We further assessed the cell potential using Pd, which had not been preloaded with hydrogen. The resulting cell potential appeared between those of Pt and H‐Pd (Figure 1d and Table S1). In the absence of pre‐stored hydrogen within the Pd lattice, Pd initially behaves as a Pt foil, resulting in a higher cell potential than H‐Pd. Similar cell potential trends were observed in electrolytes with different proton concentrations (0.1 M phosphate buffer, and 0.1 M KOH), confirming the robustness of the mechanism (Figure S4).

To further substantiate the mechanistic interpretation described above, we deconvoluted the cell potential distribution across the electrodes and the membrane by resolving the potential at each membrane–electrolyte and electrode–electrolyte interface [32]. (Figure 2, Figures S5 and S6, and Tables S2, S3) The potential profile could be broken down into six distinct regions, corresponding to: (1) the cathodic HER potential, (2) ionic resistance within the catholyte, (3) the oxidation potential at the catholyte‐facing membrane surface, (4) the reduction potential at the anolyte‐facing membrane surface, (5) ionic resistance within the anolyte, and (6) the anodic OER potential.

**FIGURE 2:**
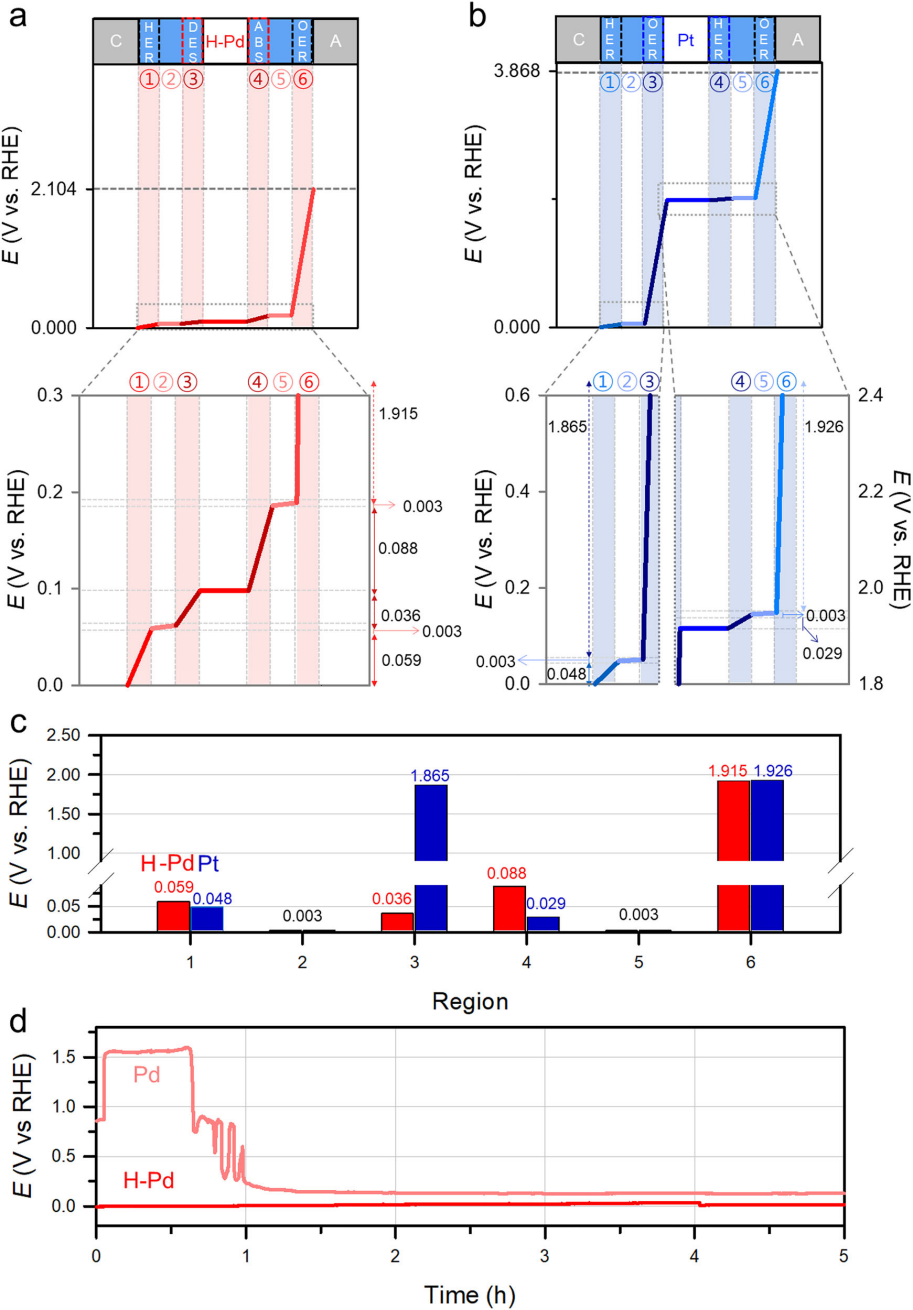
Local membrane potentials measured across H‐Pd and Pt. Position‐resolved potentials measured on the (a) H‐Pd and (b) Pt under −1 mA cm^−2^. (c) Potential comparison graph for each region. (d) membrane potential changes in region 3 for pristine Pd and H‐Pd under −1 mA cm^−2^ for 5 h.

A comparison between H‐Pd (Figure 2a) and Pt (Figure 2b) revealed that the regions (1), (2), (4), (5), and (6) exhibited similar potential drops irrespective of membrane composition, whereas region (3) displayed a pronounced difference (Figure 2c). For H‐Pd, proton desorption, occurring near 0 V vs. RHE (revesible hydrogen electrode) [25], dominated the oxidation process, affording a membrane potential of 0.036 V (vs. RHE). In contrast, for Pt, lacking hydrogen‐storage capacity, OER participated in the oxidation to sustain charge transfer, resulting in a markedly higher potential of 1.865 V (vs. RHE). This difference confirms that the fundamental shifts in the preferential interfacial bipolar electrochemical reactions enable proton transport through H‐Pd, but not through Pt. Although region 4 is expected to involve competitive proton absorption for H‐Pd, whereas only HER occurs for Pt, the thermodynamic potentials for these processes are similar [33], so that the resulting potential differences are not notable in this region.

We further monitored the membrane oxidation potential (region 3) under continuous current for 5 h using Pd in its hydrogen‐free state (Figure 2d). Initially, OER governed the interfacial process, resulting in a high oxidation potential of 1.560 V (vs. RHE). Over time, however, as hydrogen gradually accumulated within the Pd lattice and proton desorption began to participate in the surface bipolar electrochemical oxidation process, the interfacial potential progressively decreased to 0.126 V (vs. RHE) after 5 h. In contrast, when H‐Pd was used from the outset, the potential remained constant throughout the experiment. Collectively, these results verify that proton transport is enabled by a unique bipolar electrochemical proton‐pumping mechanism in the bipolar H‐Pd membrane.

To provide direct evidence for the bipolar electrochemical proton‐pumping mechanism, we performed isotope‐labeling experiments with reference to the approach reported by Ye et al. [14]. Proton transport through the Pd membrane was verified by monitoring the increase in the ^1^H NMR signal corresponding to H_2_O in a D_2_O‐based 0.1 M NaClO_4_ catholyte during electrolysis with an H_2_O‐based 0.1 M NaClO_4_ anolyte. Upon applying a current density of 13.3 mA cm^−2^ for 2 h, the H_2_O concentration in D_2_O‐based catholyte increased from 104.3 to 148.8 mM (Figure 3). Extending the electrolysis time to 20 h under the same current density further increased the H_2_O concentration to 495.5 mM (Figure 3). The nearly 9‐fold increase in the H_2_O signal with the tenfold increase in the total passed charge provides direct evidence for electrochemically driven proton pumping through the Pd membrane from anolyte to catholyte. We estimate that approximately 10% of the applied charge was consumed by the OER at the catholyte‐facing side of Pd, leading to a partial loss in the H‐signal increase. This issue is discussed in detail in the following section. For comparison, when the same cell configuration was maintained under open‐circuit potential conditions for 20 h without applying any current, the H_2_O concentration in D_2_O remained almost unchanged at 118.5 mM (Figure 3), implying that passive H_2_O crossover by diffusion was negligible and convincing the bipolar electrochemical proton transport through Pd.

**FIGURE 3:**
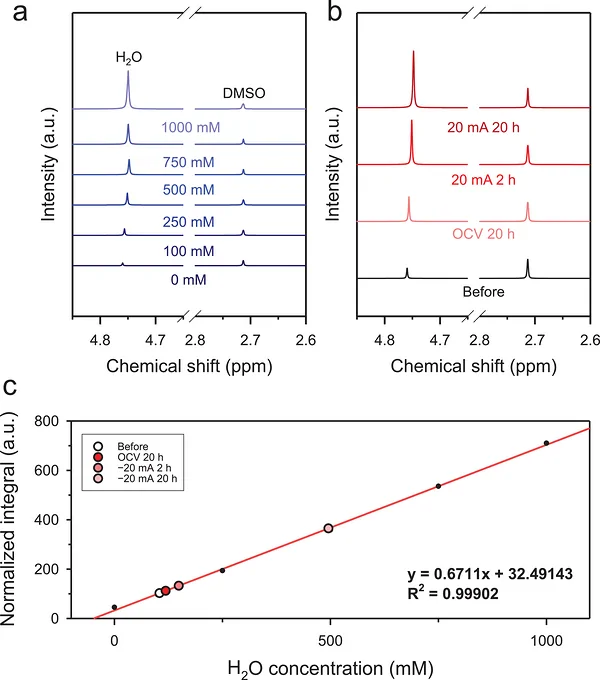
^1^H NMR calibration for H_2_O quantification in D_2_O. (a) ^1^H NMR spectra of calibration standards containing different concentrations of H_2_O in D_2_O. DMSO was used as the internal standard, and the spectra were normalized to the DMSO singlet at ∼2.72 ppm. The H_2_O/HOD resonance appears at ∼4.75 ppm. (b) ^1^H NMR spectra of the D_2_O catholyte collected before operation, after 20 h at OCV, after electrolysis at −20 mA for 2 h, and after electrolysis at −20 mA for 20 h. DMSO was used as the internal standard. (c) Calibration curve constructed from the normalized integral of the H_2_O/HOD resonance as a function of H_2_O concentration.

We next evaluated the proton transport efficiency of the metal membranes by inducing competitive hydrazine oxidation (N_2_H_4_ + 4OH^−^ → N_2_ + 4H_2_O + 4e^−^, *E* ^0^ = −0.33 V vs. RHE) at the catholyte‐facing interface (Figure 4a). Since hydrazine oxidation proceeds in a potential region similar to that of proton desorption [34] and hydrazine can be accurately quantified [35], we employed hydrazine oxidation as a competitive reaction to assess proton transport efficiency. 50 mM N_2_H_4_ + 15 mM KOH and 0.1 M HClO_4_ were used as the catholyte and anolyte, respectively. A Pt wire and a dimensionally stable anode (DSA) served as the cathode and anode, respectively, and a constant current density of −6.5 mA cm^−2^ was applied for 1 h. By comparing the hydrazine concentration in the catholyte before and after electrolysis using ion chromatography (IC), we determined the fraction of the applied charge consumed by hydrazine oxidation and proton transport efficiency. We note that in the present system, OER could also potentially occur as an additional competing reaction. To examine this possibility, we analyzed the gaseous O_2_ product using gas chromatography (GC), but the O_2_ signal was below the detection limit (Figure S7), indicating that the contribution of OER was negligible. Therefore, OER was excluded from the subsequent charge‐balance analysis.

**FIGURE 4:**
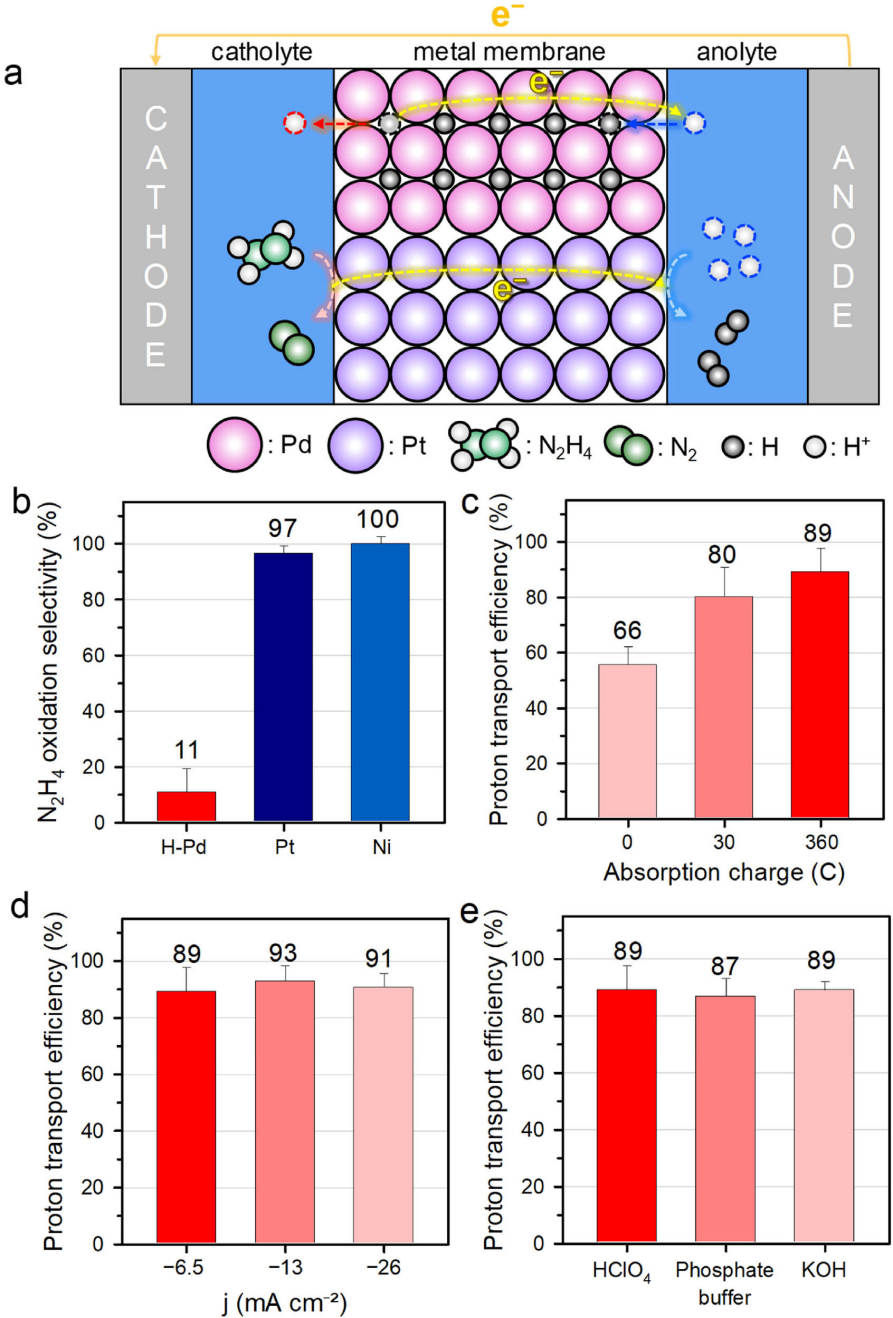
Proton transport efficiency of bipolar metal membranes. (a) Schematic illustration showing the competition between proton desorption and N_2_H_4_ oxidation and proton absorption and HER at the membrane–electrolyte interfaces. (b) N_2_H_4_ oxidation selectivity at catholyte‐facing interfaces of metal foils. Proton transport efficiency of H‐Pd as a function of (c) proton absorption charge, (d) current density, and (e) anolyte pH.

When Pt and Ni were used as the membrane (Figure 4b), both metals exhibited nearly 100% selectivity toward hydrazine oxidation at the catholyte‐facing interface, corroborating their inability to transport protons across the membrane. In contrast, the proton‐transport efficiency of Pd strongly depended on the extent of electrochemical hydrogen pre‐loading (Figure 4c). When Pd was used without any prior hydrogen loading, the proton transport efficiency was only 66%. Pre‐loading with 30 C of charge increased the efficiency to 80%, whereas sufficient pre‐loading with 360 C, which is above the theoretical hydrogen‐storage capacity [36], further raised the efficiency to 89% under the competing reaction condition. This dependence on the internal hydrogen concentration indicates that hydrogen atom diffusion through the Pd/PdH*_x_* lattice is a key rate‐determining step in proton transport.

H‐Pd maintained high proton‐transport efficiencies even at elevated current densities (Figure 4d). When −13 and −26 mA cm^−2^ were applied, the coulombic efficiency for the proton transport reached 93% and 91%, respectively, demonstrating that H‐Pd can sustain proton transport even under substantial proton flux. To test the practical applicability of Pd membrane in high current density regime, we further applied −100 mA cm^−2^. As a result, the proton transport efficiency decreased to 86% at −100 mA cm^−2^ (Figure S8). At high current density regime, the hydrogen concentration in the Pd lattice near the catholyte‐facing interface may become depleted, which can promote the competing hydrazine oxidation reaction, lowering the proton transport efficiency.

Theoretically, assuming full occupation of hydrogen in the octahedral interstitial sites at the anolyte‐facing side of Pd (PdH_1_) and complete hydrogen depletion at the catholyte‐facing side of Pd, limiting current density is ∼ 140 mA cm^−2^ (see Supporting Information), but it is difficult to achieve such a maximum gradient because hydrogen cannot readily occupy all Pd lattice sites at a 1:1 H/Pd ratio. In addition, lattice distortion and associated diffusion resistance may further limit hydrogen diffusion. Therefore, at high current densities, hydrogen diffusion through the Pd lattice may not fully keep the pace with the interfacial charge‐transfer rate. This can lead to hydrogen depletion near the catholyte‐facing Pd surface, increased charge consumption by competing electrolyte oxidation reactions, and consequently a lower proton transport efficiency. One possible strategy to increase the achievable current density is to reduce the Pd membrane thickness, thereby shortening the hydrogen diffusion length. However, this approach may compromise the mechanical durability of the membrane. Therefore, future work should focus on developing thin Pd‐based membranes that maintain sufficient mechanical robustness while enabling faster hydrogen diffusion. Proton‐transport efficiency remained high (∼89%) even when the anolyte pH was increased on the proton‐absorption side (Figure 4e), indicating the efficient proton uptake in H‐Pd even at lower proton concentrations.

In addition, we evaluated the proton transport efficiency at the anolyte‐facing side of the Pd membrane in 0.1 M HClO_4_ electrolyte. At this interface, bipolar electrochemical reduction processes occur, and the possible reduction reactions include hydrogen evolution (2H^+^ + 2e^−^ → H_2_) and hydrogen absorption into Pd (Pd + *x*H^+^ + *x*e^−^ → PdH*_x_*). In a manner analogous to the analysis described above, we quantified the gaseous H_2_ product from hydrogen evolution using GC and then back‐calculated the extent of hydrogen absorption into Pd. As a result, hydrogen evolution accounted for below 5% of the total current regardless of the degree of hydrogen pre‐charging and current density (Figure S9), indicating the proton transport efficiency at the anolyte‐facing side of the Pd was above 95%. These results show that proton transport efficiency at the catholyte‐facing Pd side has a more pronounced influence on the overall proton transport efficiency. Furthermore, the charge corresponding to hydrogen absorption into Pd at the anolyte‐facing side exceeded the charge attributable to PdH*_x_* oxidation at the catholyte‐facing side. This charge imbalance indicates that not all absorbed hydrogen participates in electrochemical proton transport, suggesting that a fraction of the lattice hydrogen is released as H_2_ rather than being oxidized to protons. Overall, these findings suggest that improving the proton transport efficiency requires interfacial modulation at the catholyte‐facing Pd side to promote faster and more selective PdH*_x_* oxidation.

Finally, quantitative analysis of hydrazine in the anolyte revealed no detectable hydrazine crossover within the IC detection limit of 500 µM (Figure S10), demonstrating the strong molecular‐blocking capability of the dense bipolar H‐Pd membrane. Taken together, these results demonstrate that H‐Pd inherently functions as a truly crossover‐impervious proton‐transporting membrane, and its proton‐transport capability arises from bipolar electrochemical proton pumping coupled with solid‐state hydrogen diffusion in the PdH*_x_* lattice.

Next, we demonstrate the practical use of the bipolar H‐Pd membrane in a Li‐NRR coupled with the OER for NH_3_ synthesis directly using N_2_ and H_2_O as reactants. Our experimental setup was adapted from the “continuous‐flow system” developed by Fu et al. (Figures S11 and S12) [37] and is modified to incorporate the bipolar H‐Pd membrane (Figures S13 and S14) and an OER anode in place of the H_2_ oxidation reaction (HOR) anode. A stainless‐steel cloth (SSC) gas‐diffusion electrode (GDE) is used as the cathode, supplied with N_2_ to drive Li‐NRR, while IrO_2_ is loaded on the anode to catalyze the OER (details are provided in Supporting Information). Unless otherwise noted, the catholyte consists of 50 mL of tetrahydrofuran (THF) containing 1 M LiBF_4_ and 43 mM ethanol (EtOH) as the proton carrier, and the anolyte is 0.1 M phosphate buffer solution. Electrochemical operation is initiated by connecting the Pd membrane and the anode in a two‐electrode configuration to pre‐load hydrogen in the Pd membrane (23 376 C cm^−3^; Figure S15). The cell is then switched to a three‐electrode configuration using a Pt wire in the catholyte as a pseudo‐reference electrode, and Li‐NRR is performed by chronopotentiometry (CP) at −3 mA cm^−2^ for 2 h. In the H‐Pd membrane configuration, the Pd foil is assembled to separate the aqueous anolyte from the non‐aqueous catholyte, while each electrolyte is continuously recirculated. Protons generated by the OER at the anode are transported across the bipolar H‐Pd membrane into the catholyte, where they are shuttled by a proton carrier and consumed in Li‐NRR (Figure 5a).

**FIGURE 5:**
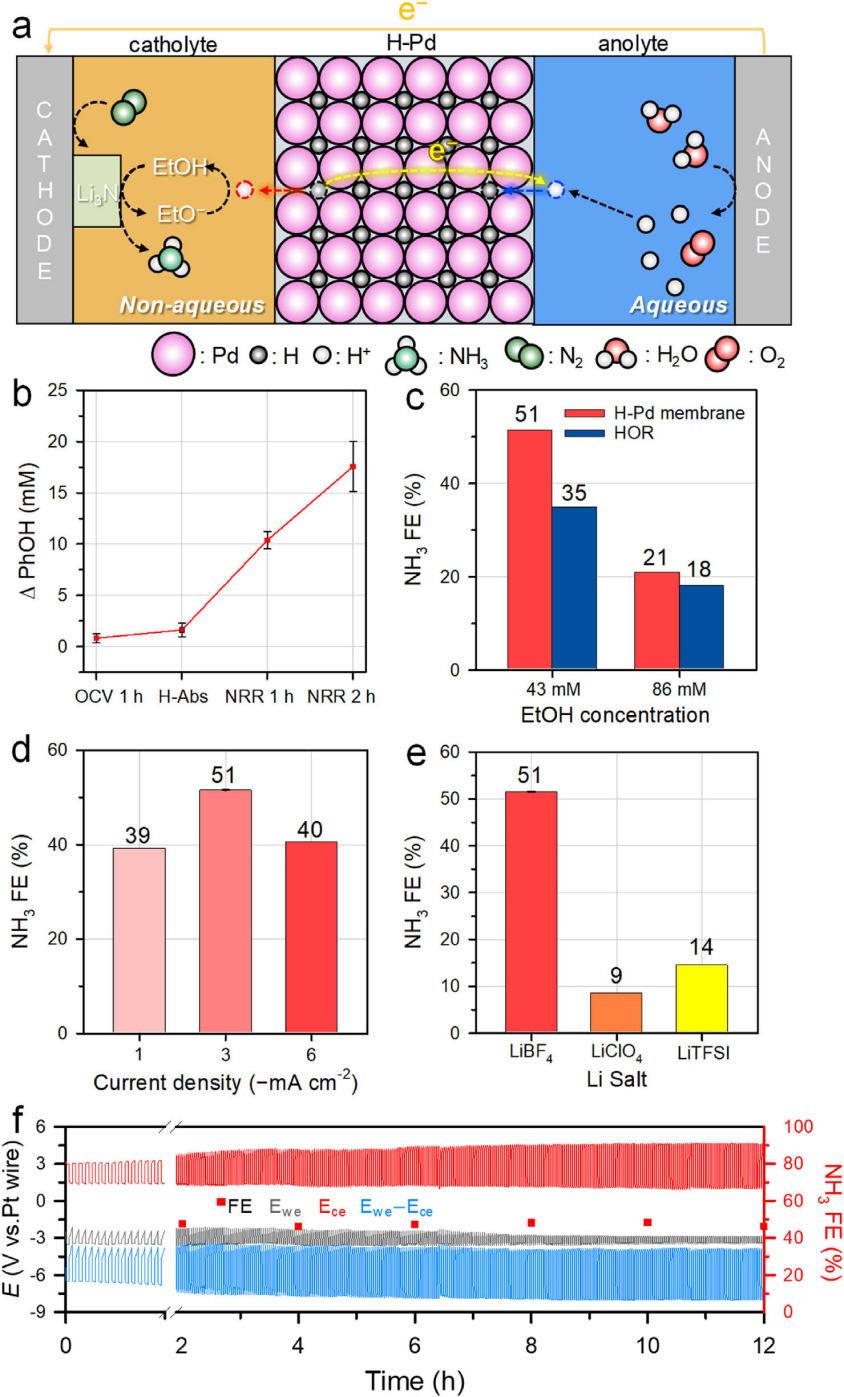
Li‐NRR coupled with OER using the bipolar H‐Pd membrane. (a) Schematic illustration of the non‐aqueous Li‐NRR integrating a bipolar H‐Pd membrane to couple an aqueous anolyte while suppressing water crossover. (b) Validation of proton transport using a phenoxide (PhO^−^) proton acceptor: increase in PhOH concentration relative to the initial state (ΔPhOH) at each condition, after open‐circuit voltage operation (OCV 1 h), hydrogen absorption in the Pd membrane (H‐abs), and Li‐NRR for 1 and 2 h. (c) NH_3_ FEs with the bipolar H‐Pd membrane/H_2_O configuration and the HOR configuration under EtOH concentrations of 43 and 86 mM. (d) Dependence of NH_3_ FE on applied current density. (e) NH_3_ FEs under different Li salts. (f) Long‐term stability test operated under a potential cycling (1 min at CP −3 mA cm^−2^ followed by 1 min at 0 mA cm^−2^) for 12 h. The anode, cathode, and cell potential are shown (left axis), together with NH_3_ FE measured every 2 h (red squares; right axis). The effective areas of the electrodes and the bipolar H‐Pd membrane are 4 cm^2^.

To confirm that the H‐Pd membrane can serve as a proton transport membrane and the transported protons are subsequently consumed in NH_3_ synthesis in an aprotic catholyte, we first employed phenoxide (PhO^−^), a proton acceptor known to exhibit (electro)chemical stability under Li‐NRR conditions [38]. Accordingly, an increase in the phenol (PhOH) concentration indicates the proton transfer to PhO^−^ across the H‐Pd membrane. PhO^−^ is also analytically advantageous because PhOH and PhO^−^ can serve as references and provide well‐resolved ^1^H nuclear magnetic resonance (NMR) signals, enabling straightforward quantification of protonation. For this purpose, 100 mM of lithium phenoxide was included, and the evolution of PhO^−^, PhOH, and NH_3_ was quantified to track the reaction (Figures S16 and S17). To confirm that no protons enter the catholyte from outside the electrochemical cell, the system was first operated at the open‐circuit voltage (OCV) condition for 1 h, followed by hydrogen absorption (H‐Abs) into the bipolar Pd membrane. During both steps, the PhOH concentrations changed only marginally, which were attributed to slight THF evaporation (solvent loss) rather than proton influx (Figure 5b and Figure S18). Once Li‐NRR was initiated, the PhOH concentration noticeably increased by 9.18 mM along with the NH_3_ production (8.6 µmol) after 1h of electrolysis. Its pronounced increase was also observed as 2 h of Li‐NRR elapsed. These results verify that protons are supplied to the aprotic electrolyte via the bipolar H‐Pd membrane and are channeled into the Li‐NRR.

Next, we further evaluated Li‐NRR performance for NH_3_ production at various operating conditions using EtOH, a widely used proton carrier. NH_3_ quantification was performed using ^1^H NMR spectroscopy and IC (Figures S19 and S20). Specifically, we compared two different configurations: (i) the bipolar H‐Pd membrane configuration (“H‐Pd”), and (ii) the configuration in which a Pt mesh anode catalyzes HOR and directly supplies protons to the catholyte without any membrane (“HOR”; Figure 5c). At 43 mM EtOH (0.25 vol%), the NH_3_ FE was higher than that obtained at 86 mM EtOH (0.5 vol%). Notably, the bipolar H‐Pd membrane configuration outperformed the HOR configuration at both EtOH concentrations. Based on previous studies, this difference could be attributed to H_2_ permeation through the GDE in the HOR configuration, which may lead to parasitic reactions with Li metal [39]. The cell potential for the bipolar H‐Pd membrane configuration is ∼2 V higher than that of the HOR configuration, reflecting the thermodynamic and overpotential difference between OER and HOR at the anode (Figure S21). In the HOR case, however, an additional increase in cell potential is observed during Li‐NRR operation because the side reaction at the Pt mesh anode, including the oxidation of the non‐aqueous solvent (THF), can cause surface poisoning and a larger overpotential over time [37]. Increasing the Pd membrane thickness to 100 µm did not lead to noticeable changes in Li‐NRR potential or NH_3_ FE compared with a 25 µm Pd membrane (Figure S22), supporting the effective proton transfer across the bipolar H‐Pd membrane owing to its fast hydrogen diffusion.

Figure 5d shows the dependence of FE on the applied current density for the Li‐NRR, which reaches a maximum at −3 mA cm^−2^. At higher current density (−6 mA cm^−2^), FE decreases, due to lithium over‐deposition and the associated charge losses (Figure S23b). At lower current density (−1 mA cm^−2^), the FE also decreases because of increased non‐productive charge consumption during the initial formation of the SEI and/or the development of an SEI that is less favorable for Li‐NRR under these conditions. Further optimization of the Li‐NRR condition can increase NH_3_ production with the bipolar H‐Pd membrane. Using acidic (0.1 M HClO_4_) or alkaline (0.1 M KOH) anolytes instead of 0.1 M phosphate buffer results in similar NH_3_ FEs. (Figure S24). This trend correlates with the pH‐independent proton transport efficiency of the bipolar H‐Pd membrane observed in Figure 4e, which suggests that proton delivery across the bipolar H‐Pd membrane remains largely insensitive to anolyte pH within this range, exhibiting a wide operating window.

In addition to LiBF_4_, we tested LiClO_4_ and lithium bis(trifluoromethylsulfonyl)imide (LiTFSI), which are commonly used Li salts in Li‐NRR (Figure 5e). Consistent with prior reports [40], we observed lower NH_3_ production with ClO_4_^−^ and TFSI^−^ anions, which can interact with protons and catalyze solvent degradation, including THF polymerization—changes in the electrolyte were also observed (Figure S25d,e). The post‐reaction of the cathode shows more pronounced residual Li deposits and a visibly thicker passivation layer in LiClO_4_ and LiTFSI‐based electrolytes than in LiBF_4_ (Figure S23c,d). In both cases, the NH_3_ FE is markedly lower than LiBF_4_, indicating that these salts are significantly less suitable for our system. Based on these screening results, we conducted a 12 h cycling test using a potential‐cycling strategy (1 min at CP −3 mA cm^−2^ followed by 1 min at 0 mA cm^−2^) designed to enhance both the stability and efficiency of the system [37, 41]. The cell operated stably for total 12 h (Figure 5f and Figure S26), maintaining NH_3_ FE over the entire period. No appreciable electrolyte oxidation or degradation was observed over this period as well (Figure S25f). Remarkably, even after 12 h of operation, the water content, which was measured by Karl Fischer titration showing a detection range from 10 ppm to 1%, in the catholyte remained at a negligible level of 105 ppm, known to be within the stable operation ranges. This level is much lower than the crossover values previously reported employing IEMs (Table S4). This underscores the excellent suppression of water crossover afforded by the bipolar H‐Pd membrane.

To further assess the performance of the bipolar H‐Pd membrane under extended operating conditions, we performed an extended operation using a diglyme (DG)‐based catholyte, as DG is less volatile than THF and has been employed as a more stable solvent for prolonged Li‐NRR operation [10]. The extended operation was conducted using 1 M LiBF_4_ and 43 mM EtOH in DG as the catholyte (100 mL), 0.1 M phosphate buffer as the anolyte, and a 100 µm‐thick Pd foil as the membrane. The cell was operated under controlled cycling, consisting of 1 min of CP at −3 mA cm^−2^ followed by resting at 0 mA cm^−2^ until the cathode potential reached −2.3 V vs. Pt wire. Under these conditions, the cell sustained operation for approximately 79 h, with NH_3_ FE remaining at 50% even after 72 h, as calculated from the total amount of NH_3_ quantified in both the catholyte and acid trap (Figure S27). The operation was eventually terminated due to catholyte gelation, which blocked the electrolyte flow channel. Post‐operation ^1^H NMR analysis of the catholyte showed additional resonances near 3.2 ppm, indicating DG‐derived decomposition or the formation of oligomeric species (Figure S28).

Post‐mortem characterization of the Pd membrane was then performed to assess membrane degradation and PdH_x_ phase stability. XRD analysis of the catholyte‐facing side confirmed that the hydrogenated PdH_x_ phase remained the dominant crystalline phase after the extended operation, although weak metallic Pd peaks were also observed (Figure S29), indicating that the Pd membrane retained the hydrogenated phase necessary for lattice‐mediated proton transport during prolonged operation. Scanning electron microscopy (SEM) images further revealed that the aqueous anolyte‐facing side retained a relatively smooth morphology, whereas the catholyte‐facing side exhibited region‐dependent roughened surface changes (Figure S30). SEM–energy‐dispersive X‐ray spectroscopy (SEM–EDS) analysis showed the presence of C‐ and O‐containing species, suggesting that these morphological changes were associated with electrolyte‐derived deposits and degradation products (Figure S31). In addition, ICP–OES analysis revealed that the catholyte contained approximately 5.0 ppm of dissolved Pd after extended operation, indicating partial dissolution of Pd (Figure S32). Collectively, these results suggest that the bipolar H‐Pd membrane sustains PdH_x_‐mediated proton transport for a substantially longer period than the 12 h cycling test, while revealing catholyte gelation, catholyte‐facing surface degradation, and partial Pd dissolution as current limitations of the present system. Addressing these challenges will require coordinated advances in electrolyte formulation, flow‐cell engineering, and Pd membrane stabilization strategies.

Finally, based on the optimized water‐fed Li‐NRR configuration, we calculated the full‐cell energy efficiency to be 10.1% (Figure S33). Benchmarking against prior Li‐NRR studies that couple an OER to a non‐aqueous catholyte shows that our system exhibits superior performance under 1 bar conditions, representing one of the highest reported values at ambient pressure and in a continuous‐flow system with an N_2_‐gas‐fed electrolyzer.

## 3 Conclusions

This work establishes a fundamentally distinct proton‐transport mechanism using the bipolar electrochemical H‐Pd membrane, enabling selective proton transport while fully suppressing undesirable crossover. The resolved potential profile reveals that reversible proton uptake and release on the H‐Pd interface induce bipolar electrochemical proton transfer at the membrane–electrolyte interfaces, which is unattainable in Pt or Ni evolving electrolyte oxidation and reduction. The bipolar H‐Pd membrane enables coupling of a non‐aqueous catholyte for the Li‐NRR with an aqueous anolyte for the OER, allowing H_2_O to serve as the proton source without destabilizing the Li chemistry. We achieved an NH_3_ FE of 51% in the hybrid electrolyte system and sustained stable operation for 12 h in a continuous‐flow electrolyzer with negligible water crossover across the bipolar H‐Pd membrane. We envisage that these findings show that the lattice‐channeled ion‐pumping pathway of the bipolar metal membranes can overcome the long‐standing conductivity–selectivity constraint that limits polymer‐based membranes and open a new design space for electrochemical systems that require strict compartmentalization, including Li‐mediated NH_3_ synthesis, membrane‐separated electrosynthesis, and next‐generation energy‐conversion platforms.

## Author Contributions

**Jiyeon Baek**: formal analysis, investigation, visualization, methodology, writing – original draft, data curation. **Yeongbae Jeon**: writing – original draft, data curation, formal analysis, investigation, validation. **Seunga Lee**: investigation, data curation, formal analysis. **Ahee Choi**: data curation, investigation, formal analysis. **Joonmok Shim**: investigation. **Sun Hyung Kim**: investigation. **Ho Woun Jung**: investigation. **Kyungho Lee**: investigation. **Churl Hee Cho**: supervision, writing – review and editing. **Hyung Chul Yoon**: supervision, writing – review and editing, funding acquisition. **Yun Jeong Hwang**: supervision, writing – review and editing, investigation, conceptualization, methodology, validation. **Jae Hyung Kim**: conceptualization, methodology, data curation, supervision, investigation, formal analysis, visualization, writing – original draft, writing – review and editing, validation.

## Conflicts of Interest

The authors declare no conflicts of interest.

## Supporting information




**Supporting File**: advs76415‐sup‐0001‐SuppMat.docx.

## Data Availability

The data that support the findings of this study are available from the corresponding author upon reasonable request.

## References

[1] S. Hu , M. Lozada‐Hidalgo , F. C. Wang , et al., “Proton Transport Through One‐Atom‐Thick Crystals,” Nature 516 (2014): 227–230, 10.1038/nature14015. 25470058

[2] A. Wang , C. Breakwell , F. Foglia , et al., “Selective Ion Transport Through Hydrated Micropores in Polymer Membranes,” Nature 635 (2024): 353–358, 10.1038/s41586-024-08140-2. PMC11560840 39506120

[3] C. Dai , Q. Wu , T. Wu , et al., “Suppressing Product Crossover and C–C Bond Cleavage in a Glycerol Membrane Electrode Assembly Reformer,” Energy & Environmental Science 17 (2024): 6350–6359, 10.1039/D4EE01824A.

[4] E. W. Lees , J. C. Bui , O. Romiluyi , A. T. Bell , and A. Z. Weber , “Exploring CO_2_ Reduction and Crossover in Membrane Electrode Assemblies,” Nature Chemical Engineering 1 (2024): 340–353, 10.1038/s44286-024-00062-0.

[5] C. R. Wang , J. M. Stansberry , R. Mukundan , et al., “Proton Exchange Membrane (PEM) Water Electrolysis: Cell‐Level Considerations for Gigawatt‐Scale Deployment,” Chemical Reviews 125 (2025): 1257–1302, 10.1021/acs.chemrev.3c00904. PMC11996138 39899322

[6] Y. Zhang , Y. Yang , D. Ye , et al., “Hydrogen Crossover Raises Serious Concerns on Proton Exchange Membrane Water Electrolyzer,” The Innovation 7 (2026): 101089, 10.1016/j.xinn.2025.101089. PMC13237848 42254960

[7] M. L. Perry , J. D. Saraidaridis , and R. M. Darling , “Crossover Mitigation Strategies for Redox‐Flow Batteries,” Current Opinion in Electrochemistry 21 (2020): 311–318, 10.1016/j.coelec.2020.03.024.

[8] N. Lazouski , M. Chung , K. Williams , M. L. Gala , and K. Manthiram , “Non‐Aqueous Gas Diffusion Electrodes for Rapid Ammonia Synthesis From Nitrogen and Water‐Splitting‐Derived Hydrogen,” Nature Catalysis 3 (2020): 463–469, 10.1038/s41929-020-0455-8.

[9] H.‐L. Du , M. Chatti , R. Y. Hodgetts , et al., “Electroreduction of Nitrogen With Almost 100% Current‐to‐Ammonia Efficiency,” Nature 609 (2022): 722–727, 10.1038/s41586-022-05108-y. 35868345

[10] S. Li , Y. Zhou , X. Fu , et al., “Long‐Term Continuous Ammonia Electrosynthesis,” Nature 629 (2024): 92–97, 10.1038/s41586-024-07276-5. 38503346

[11] J. H. Kim , J.‐E. Cha , H. Ju , et al., “Utilizing Water as a Proton Source for Sustainable Li‐Mediated Electrochemical Ammonia Synthesis,” Chemical Engineering Journal 497 (2024): 154644, 10.1016/j.cej.2024.154644.

[12] J. Miao , L. Wang , Y. Zhao , X. Chen , H. Li , and Y. Shao‐Horn , “Harnessing Lithium‐Mediated Green Ammonia Synthesis With Water Electrolysis Boosted by Membrane Electrolyzer With Polyoxometalate Proton Shuttles,” Angewandte Chemie International Edition 64 (2025): 202503465, 10.1002/anie.202503465. 40289915

[13] H. Bemana , H. Schumann , M. McKee , et al., “Accelerating Lithium‐Mediated Nitrogen Reduction Through an Integrated Palladium Membrane Hydrogenation Reactor,” Nature Communications 16 (2025): 6696, 10.1038/s41467-025-62088-z. PMC12304132 40721605

[14] B. Ye , C. Burdis , V. Mints , et al., “Continuous Ammonia Electrosynthesis From Nitrogen and Water in a Monolithic Pd Membrane‐Based Flow Cell,” ACS Energy Letters 11 (2026): 1907–1915, 10.1021/acsenergylett.5c03617. PMC12910716 41710781

[15] A. Tsuneto , A. Kudo , and T. Sakata , “Lithium‐mediated Electrochemical Reduction of High Pressure N_2_ to NH_3_ ,” Journal of Electroanalytical Chemistry 367 (1994): 183–188, 10.1016/0022-0728(93)03025-K.

[16] M. Spry , O. Westhead , R. Tort , et al., “Water Increases the Faradaic Selectivity of Li‐Mediated Nitrogen Reduction,” ACS Energy Letters 8 (2023): 1230–1235, 10.1021/acsenergylett.2c02792. PMC9926485 36816776

[17] M. Spry , J. Rietbrock , O. Westhead , et al., “In Situ Spectroscopy Reveals How Water‐Driven SEI Formation Controls Selectivity in Li‐Mediated N_2_ Reduction,” Energy & Environmental Science 18 (2025): 8414–8429, 10.1039/D5EE01961C.

[18] D. Chen , S. Wang , M. Xiao , and Y. Meng , “Synthesis and Characterization of Novel Sulfonated Poly(Arylene Thioether) Ionomers for Vanadium Redox Flow Battery Applications,” Energy & Environmental Science 3 (2010): 622–628, 10.1039/B917117G.

[19] S. P. Jiang , Z. Liu , and Z. Q. Tian , “Layer‐by‐Layer Self‐Assembly of Composite Polyelectrolyte–Nafion Membranes for Direct Methanol Fuel Cells,” Advanced Materials 18 (2006): 1068–1072, 10.1002/adma.200502462.

[20] K.‐D. Kreuer , “Proton Conductivity: Materials and Applications,” Chemistry of Materials 8 (1996): 610–641, 10.1021/cm950192a.

[21] H. B. Park , J. Kamcev , L. M. Robeson , M. Elimelech , and B. D. Freeman , “Maximizing the Right Stuff: The Trade‐Off Between Membrane Permeability and Selectivity,” Science 356 (2017): aab0530, 10.1126/science.aab0530. 28619885

[22] G. M. Geise , M. A. Hickner , and B. E. Logan , “Ionic Resistance and Permselectivity Tradeoffs in Anion Exchange Membranes,” ACS Applied Materials & Interfaces 5 (2013): 10294–10301, 10.1021/am403207w. 24040962

[23] D. Kitto and J. Kamcev , “Predicting the Conductivity–Selectivity Trade‐Off and Upper Bound in Ion‐Exchange Membranes,” ACS Energy Letters 9 (2024): 1346–1352, 10.1021/acsenergylett.4c00301.

[24] S. E. Fosdick , K. N. Knust , K. Scida , and R. M. Crooks , “Bipolar Electrochemistry,” Angewandte Chemie International Edition 52 (2013): 10438–10456, 10.1002/anie.201300947. 23843205

[25] R. S. Sherbo , M. Moreno‐Gonzalez , N. J. J. Johnson , D. J. Dvorak , D. K. Fork , and C. P. Berlinguette , “Accurate Coulometric Quantification of Hydrogen Absorption in Palladium Nanoparticles and Thin Films,” Chemistry of Materials 30 (2018): 3963–3970, 10.1021/acs.chemmater.8b01324.

[26] M. A. V. Devanathan and Z. Stachurski , “The Adsorption and Diffusion of Electrolytic Hydrogen in Palladium,” Proceedings of the Royal Society of London Series A Mathematical and Physical Sciences 270 (1962): 90–102, 10.1098/rspa.1962.0205.

[27] C.‐C. Hu and T.‐C. Wen , “Voltammetric Investigation of Hydrogen Sorption/Desorption at/Within Oxide‐Derived Pd Electrodes in NaOH and H_2_SO_4_ ,” Journal of the Electrochemical Society 141 (1994): 2996–3001, 10.1149/1.2059271.

[28] J. P. Burger , S. Senoussi , and B. Soufaché , “Electrical and Magnetic Properties of Palladium Hydrides Compared With Those of Pure Palladium,” Journal of the Less Common Metals 49 (1976): 213–222, 10.1016/0022-5088(76)90036-9.

[29] N. J. J. Johnson , B. Lam , B. P. MacLeod , et al., “Facets and Vertices Regulate Hydrogen Uptake and Release in Palladium Nanocrystals,” Nature Materials 18 (2019): 454–458, 10.1038/s41563-019-0308-5. 30858567

[30] R. M. Crooks , “Principles of Bipolar Electrochemistry,” ChemElectroChem 3 (2016): 357–359, 10.1002/celc.201500549.

[31] T. F. Jaramillo , K. P. Jørgensen , J. Bonde , et al., “Trends in the Exchange Current for Hydrogen Evolution,” Journal of the Electrochemical Society 152 (2005): J23–J26, 10.1149/1.1856988.

[32] E. Villani and S. Inagi , “Experimental Methods for Measuring Potential and Current Density Distributions at Bipolar Electrodes,” Analytical Chemistry 97 (2025): 5837–5846, 10.1021/acs.analchem.4c05641. PMC11948184 40084680

[33] M. Moreno‐Gonzalez , A. Huang , P. A. Schauer , et al., “Sulfuric Acid Electrolyte Impacts Palladium Chemistry at Reductive Potentials,” Chemistry of Materials 32 (2020): 9098–9106, 10.1021/acs.chemmater.0c01199.

[34] T. Y. Burshtein , Y. Yasman , L. Muñoz‐Moene , J. H. Zagal , and D. Eisenberg , “Hydrazine Oxidation Electrocatalysis,” ACS Catalysis 14 (2024): 2264–2283, 10.1021/acscatal.3c05657.

[35] M. Mori , K. Tanaka , Q. Xu , M. Ikedo , H. Taoda , and W. Hu , “Highly Sensitive Determination of Hydrazine Ion by Ion‐Exclusion Chromatography With Ion‐Exchange Enhancement of Conductivity Detection,” Journal of Chromatography A 1039 (2004): 135–139, 10.1016/j.chroma.2004.03.075. 15250415

[36] F. D. Manchester , A. San‐Martin , and J. M. Pitre , “The H‐Pd (hydrogen‐palladium) System,” Journal of Phase Equilibria 15 (1994): 62–83, 10.1007/BF02667685.

[37] X. Fu , J. B. Pedersen , Y. Zhou , et al., “Continuous‐Flow Electrosynthesis of Ammonia by Nitrogen Reduction and Hydrogen Oxidation,” Science 379 (2023): 707–712, 10.1126/science.adf4403. 36795804

[38] X. Fu , A. Xu , J. B. Pedersen , et al., “Phenol as Proton Shuttle and Buffer for Lithium‐Mediated Ammonia Electrosynthesis,” Nature Communications 15 (2024): 2417, 10.1038/s41467-024-46803-w. PMC10948763 38499554

[39] R. A. Vilá , D. T. Boyle , A. Dai , et al., “LiH Formation and Its Impact on Li Batteries Revealed by Cryogenic Electron Microscopy,” Science Advances 9 (2023): adf3609, 10.1126/sciadv.adf3609. PMC10038333 36961896

[40] X. Fu , S. Li , N. H. Deissler , J. B. V. Mygind , J. Kibsgaard , and I. Chorkendorff , “Effect of Lithium Salt on Lithium‐Mediated Ammonia Synthesis,” ACS Energy Letters 9 (2024): 3790–3795, 10.1021/acsenergylett.4c01655.

[41] S. Z. Andersen , M. J. Statt , V. J. Bukas , et al., “Increasing Stability, Efficiency, and Fundamental Understanding of Lithium‐Mediated Electrochemical Nitrogen Reduction,” Energy & Environmental Science 13 (2020): 4291–4300, 10.1039/D0EE02246B.

