# Supplementary material for: Bipolar Palladium Membrane Enabling Crossover‐Free Selective Proton Transport
Source: Adv Sci (Weinh). 2026 Jul 6;13(54):e76415. doi: 10.1002/advs.76415 (PMC13335914; doi:10.1002/advs.76415)
Supplement: Supplementary file 1 [file ADVS-13-e76415-s001.docx]

Supporting Information

**Bipolar Palladium Membrane Enabling Crossover-Free Selective Proton Transport**

**Jiyeon Baek,^1,2,4^ Yeongbae Jeon,^3,4^ Seunga Lee,^1^ Ahee Choi,^3^ Joonmok Shim,^1^ Kyungho Lee,^1^ Ho Woun Jung,^1^ Sun Hyung Kim,^1^ Churl Hee Cho,*^,2^ Hyung Chul Yoon,*^,1^ Yun Jeong Hwang,*^,3^ and Jae Hyung Kim*^,1^**

^1^Clean Fuel Research Laboratory, Korea Institute of Energy Research, 152 Gajeong-ro, Yuseong-gu, Daejeon 34129, Republic of Korea.

^2^Graduate School of Energy Science and Technology, Chungnam National University, Daejeon 34134, Republic of Korea.

^3^Department of Chemistry, College of Natural Science, Seoul National University (SNU), Seoul 08826, Republic of Korea.

^4^These authors contributed equally: Jiyeon Baek, Yeongbae Jeon.

*e-mail: kimjh1594@kier.re.kr; yjhwang1@snu.ac.kr; hyoon@kier.re.kr; choch@cnu.ac.kr;

**Table of Contents**

| **Materials and methods** |  |
| --- | --- |
| **Meterials** | **S5** |
| **Cell and membrane potential measurements in aqueous electrolytes** | **S6** |
| **Gaseous Product Analysis by GC** | **S6** |
| **Hydrazine experiments** | **S7** |
| **Hydrazine quantification** | **S7** |
| **Theoretical limiting current density** | **S8** |
| **D₂O isotope experiment and quantification of water-derived protons by ^1^H NMR** | **S9** |
| **Li-NRR with HOR experiment** | **S10** |
| **Li-NRR with bipolar H-Pd membrane experiment** | **S10** |
| **Cleaning procedures** | **S11** |
| **Preparation of anhydrous solvents** | **S11** |
| **Phenoxide proton carrier experiments** | **S11** |
| **NH_3_ quantification** | **S12** |
| **Faradaic efficiency** | **S13** |
| **Surface characterization** | **S13** |
| **Pd quantification** | **S13** |
| **Energy efficiency** | **S14** |
| **Supporting Figures, Tables, and Texts** |  |
| **Figure S1** **\|** Configuration of the electrochemical cells. | **S17** |
| **Figure S2 \|** CP for pre-hydrogen absorption. | **S18** |
| **Figure S3 \|** XRD patterns of H-Pd and Pd. | **S19** |
| **Table S1 \|** Cell potentials using different types of membranes in various electrolytes. | **S20** |
| **Figure S4 \|** Cell potentials measured using different types of membranes in neutral and alkaline electrolytes. | **S21** |
| **Figure S5 \|** Potential measurements at different regions using H-Pd membrane. | **S22** |
| **Figure S6 \|** Potential measurements at different regions using Pt foil. | **S23** |
| **Table S2 \|** Potential distribution at each region in the electrochemical cell. | **S24** |
| **Table S3 \|** Ohmic resistances of catholytes and anolytes when H-Pd and Pt were used as the membrane. | **S25** |
| **Figure S7 \|** Quantification of O_2_ gas. | **S26** |
| **Figure S8 \|** Proton transport efficiency of H-Pd membrane at a current density of −100 mA cm^−2^. | **S27** |
| **Figure S9 \|** H_2_ evolution selectivity obtained by GC analysis at the anolyte-facing side of Pd | **S28** |
| **Figure S10 \|** Quantification of hydrazine. | **S29** |
| **Figure S11** \| Schematic illustration of the Li-NRR with HOR experiment and cell assembly. | **S30** |
| **Figure S12 \|** Photograph of the experimental setup for the Li-NRR with HOR. | **S31** |
| **Figure S13 \|** Schematic illustration of the Li-NRR with a bipolar Pd membrane experiment and cell assembly. | **S32** |
| **Figure S14 \|** Photograph of experimental setup for the Li-NRR and OER, separated by a bipolar H-Pd membrane. | **S33** |
| **Figure S15 \|** Potential profiles during hydrogen charging. | **S34** |
| **Figure S16 \|** ^1^H NMR calibration of PhOH using TMB as an internal standard. | **S35** |
| **Figure S17 \|** Calibration of the indophenol blue method for ammonia quantification. | **S36** |
| **Figure S18 \|** Quantification of PhO^–^ and PhOH during phenoxide proton-carrier experiments. | **S37** |
| **Figure S19 \|** ^1^H NMR calibration of ^14^NH_4_^+^ using MA as an internal standard. | **S38** |
| **Figure S20 \|** IC calibration for NH_3_ quantification. | **S39** |
| **Figure S21 \|** Potential profiles of Li-NRR experiments with different configurations. | **S40** |
| **Figure S22 \|** Li-NRR with a bipolar Pd membrane using a 0.1 mm Pd foil. | **S41** |
| **Figure S23 \|** Photographs of SSC cathodes after Li-NRR with a bipolar Pd membrane. | **S42** |
| **Figure S24 \|** Effect of anolyte pH on NH_3_ FE in Li-NRR with bipolar Pd membrane. | **S43** |
| **Figure S25 \|** Photographs of catholytes before and after Li-NRR with a bipolar Pd membrane. | **S44** |
| **Figure S26 \|** 12 h cycling test. | **S45** |
| **Table S4 \|** Water content measured by Karl Fischer titration and references. | **S46** |
| **Figure S27 \|** Extended durability operation. | **S47** |
| **Figure S28 \|** ^1^H NMR spectra of the DG-based catholyte before and after extended durability operation. | **S48** |
| **Figure S29 \|** XRD pattern of the Pd membrane after extended durability operation. | **S49** |
| **Figure S30 \|** SEM images of the 100 μm-thick Pd foil before and after extended durability operation. | **S50** |
| **Figure S31 \|** SEM–EDS analysis of the catholyte-facing side of the Pd foil after extended durability operation. | **S51** |
| **Figure S32 \|** ICP–OES calibration curve for Pd quantification. | **S52** |
| **Figure S33 \|** Comparison of energy efficiency with literature reports. | **S53** |
|  |  |

**Methods**

**Materials**

Pd foil (0.1 mm and 0.025 mm, 99.9%), Pt foil (0.1 mm, 99.99%), Ni foil (0.1 mm, 99.95%), Iridium (IV) oxide (IrO_2_, Premion^®^, 99.99%) were purchased from Alfa Aesar. Bis(2-methoxyethyl) ether (DG, 99+%, Extra Dry, AcrosSeal^®^), dimethyl sulfoxide (DMSO, ACS, 99.9% min) were purchased from Thermo Fisher Scientific. Hydrazine monohydrate (reagent grade, 98%), potassium hydroxide (KOH, trace metals basis, 99.99%), perchloric acid (HClO_4_, trace metals basis, 70%,), potassium phosphate dibasic (K_2_HPO_4_, ACS reagent, ≥98%), potassium phosphate monobasic (KH_2_PO_4_, ReagentPlus®), methanesulfonic acid concentrate (0.1 M CH_3_SO_3_H in water (0.1N), eluent concentrate for IC), tetrahydrofuran (THF, anhydrous, ≥99.9%, inhibitor-free), ethyl alcohol (EtOH, BioUltra, absolute alcohol, without additive, ≥99.8%), ammonium chloride (NH_4_Cl, 99.998%), dimethyl sulfoxide-d_6_ (DMSO-d_6_, 99.9 atom% D), dimethyl sulfoxide-d_6_ (anhydrous DMSO-d_6_, anhydrous, 99.9 atom% D), maleic acid (MA, HO_2_CCH=CHCO_2_H, ≥99%), sulfuric acid (H_2_SO_4_, 99.999%), molecular sieves (0.4 nm, beads ~2 mm), lithium hydroxide (LiOH, reagent grade, ≥98%), acetonitrile (ACN, ACS reagent, ≥99.5%), nitric acid (HNO_3_, 0.1 M, eluent concentrate for IC), lithium phenoxide solution (PhOLi, 1.0 M in THF), 1,3,5-trimethoxybenzene (TMB, ReagentPlus^®^, ≥99%), phenol (PhOH, BioXtra, ≥99.5%), sodium hydroxide (≥97.0%), salicylic acid (C_6_H_4_(OH)COOH, ≥99.0%), sodium citrate tribasic dihydrate (C_6_H_5_Na_3_O_7_ · 2H_2_O, ≥99.0%), sodium nitroferricyanide(III) dihydrate (Na_2_[Fe(CN)_5_NO] · 2H_2_O, ≥99%), sodium hypochlorite solution (NaClO, available chlorine 4.00-4.99%), lithium perchlorate (LiClO_4_, 99.99%), Nafion™ ionomer (~5% in a mixture of lower aliphatic alcohols and water), isopropanol (IPA, 99.8%), nylon membrane filter (100 μm pore size, filter diam. 47 mm, hydrophilic), Pt wire (diam. 0.5 mm, 99.99%), Pt (Pt mesh, 100 mesh, 99.9%), Sodium perchlorate (NaClO_4_, ≥99.9%), Palladium Standard for ICP (*Trace***CERT**^®^, 1 g/L Pd in hydrochloric acid (nominal concentration)), Deuterium oxide (D_2_O, 99.9 atom% D), Deuterium oxide (D_2_O, 99.9 atom% D, contains 1 % (w/w) 3-(trimethylsilyl)-1-propanesulfonic acid, sodium salt (DSS)) were purchased from Sigma Aldrich. Acetone-d_6_ (99.9 atom% D, contains 0.05% (v/v) tetramethylsilane (TMS)) was purchased from Cambridge Isotope Laboratories. Nafion 211 was purchased by Chemours. Sulfuric acid (95.0%) and hydrogen peroxide (30.0-35.5%) for piranha solution were purchased from Samchun Chemicals. Deionized (DI) water with a resistivity of 18.2 MΩ cm was used. Ag/AgCl was purchased by ALS. Lithium tetrafluoroborate (LiBF_4_, 99.9%) was purchased from Enchem. Lithium bis(trifluoromethylsulfonyl)imide (LiTFSI, LiC_2_F_6_NO_4_S_2_, ≥99%) was purchased from Welcos Corporation. All solvents and salts related to the non-aqueous electrolyte were stored in the Ar (99.999%, KSTECH)-filled glove box (KOREA KIYON).

**Cell and membrane potential measurements in aqueous electrolytes**

Electrochemical characterizations were performed using a lab-made H-type cell (Figure S1a) in which the positions of the working and counter electrodes were fixed. Pt plates were employed as both the working electrode (WE) and counter electrode (CE). Membrane-dependent potential responses were evaluated by replacing the membrane while preserving the same electrode configuration. Nafion 211 and metal foils of Ni, Pt, and Pd (all 0.1 mm thick) were tested as the membranes. To prevent electrolyte leakage and improve sealing during cell assembly, the outer ~3 mm perimeter of each metal foil was reinforced with Be tape, and gaskets were used to secure the membrane in place. To prepare a bipolar H-Pd membrane, hydrogen was pre-loaded in Pd foils in a two-electrode system (Figure S1b): the Pd foil, electrically connected using Cu tape, served as the WE, and a dimensionally stable anode (DSA) was used as the CE. 6 mL of 0.1 M HClO_4_ was used as the electrolyte, and hydrogen absorption was carried out by applying a constant current of −100 mA for 1 h. The exposed membrane area to the electrolyte was 0.5 cm^2^. Electrochemical impedance spectroscopy (EIS) was performed at an open-circuit potential. CP measurements were conducted at −0.5 mA for 10 min to compare the electrochemical response of different membranes. Potential profiles were obtained with the same H-type cell and using two potentiostats (Figure S1c). While a constant current (−0.5 mA) was applied across the cell using the main WE and CE pair, the potential difference was measured between the electrode or membrane and Ag/AgCl reference electrode. To obtain accurate regional potentials, ohmic resistances were determined by EIS, and the potential drop from ohmic resistance was subtracted from each measured potential.

**Gaseous Product Analysis by GC**

To quantify the parasitic charge pathways, gas-phase products evolved at each compartment of the H-type cell were continuously collected and analyzed by GC. All experiments were performed under identical electrochemical conditions to those described for the competitive hydrazine oxidation experiments, using 50 mM N_2_H_4_ + 15 mM KOH as the catholyte and 0.1 M HClO_4_ as the anolyte. Gas-phase products were quantified using an Agilent 8890 GC System equipped with a thermal conductivity detector (TCD) using argon as the carrier gas. For quantification of O_2_, the catholyte compartment was sealed with gas-impermeable fittings and filled with the catholyte solution. High-purity nitrogen (99.9999%) was continuously supplied through the sealed catholyte compartment at a flow rate of 100 sccm via a mass flow controller (MFC), sweeping O_2_ evolved at the catholyte-facing membrane surface into the GC inlet, where injections were made at 3 min intervals over the course of 1 h. The O_2_ evolution selectivity was calculated as the ratio of the charge equivalent of O_2_ obtained by GC to the passed charge. For quantification of H_2_, the anolyte compartment was sealed with gas-tight fittings. N_2_ was continuously supplied to the sealed anolyte compartment at a flow rate of 10 sccm via the MFC, sweeping evolved H_2_ into the GC inlet, where injections were made at 2 min intervals over the course of 1 h. The hydrogen evolution selectivity was calculated as the ratio of the charge equivalent of H_2_ obtained by GC to the passed charge.

**Hydrazine experiments**

Hydrazine experiments were performed to evaluate proton transport efficiency. An H-type cell with separated anolyte and catholyte compartments was used (Figure S1b,d). Metal foils were applied as the membrane (area of membrane was 1.5 cm^2^). Again, the bipolar metal membrane was not connected with the potentiostat during the electrochemical step. Pd foil was subjected to sequential absorption and reaction steps, whereas other metal foils were tested only in the reaction step. The anolyte consisted of 6 mL of 0.1 M HClO_4_, while the catholyte contained 6 mL of 0.015 M KOH with 50 mM hydrazine. Hydrogen absorption was induced by applying a −100 mA current, and the charging time was controlled. After completion of the absorption step, the reaction step was performed, replacing WE from Pd into a Pt wire (Figure S1d). The solution was stirred at 500 rpm to maintain a uniform reaction environment. Hydrazine oxidation was carried out under CP conditions for 1 h under Ar-saturated conditions to remove H_2_ gas generated from the cathode, which can affect proton transport efficiency.

**Hydrazine quantification**

Hydrazine concentrations in the collected catholyte after electrolysis were quantified by IC. After electrolysis, 100 μL of the electrolyte sample was withdrawn and mixed with 900 μL of 0.1 M H_2_SO_4_ to protonate hydrazine to the hydrazinium (N_2_H_5_^+^) form and stabilize it during sample handling and IC injection. Identical acidification and dilution procedures were applied to all standards and samples to maintain a consistent analytical matrix. Hydrazine standard solutions of 0.5, 2, 4, and 8 mM were prepared in 0.1 M H_2_SO_4_ to construct calibration curves, which were used to determine hydrazine concentrations in samples. IC measurements were performed over a 15-min run time, and hydrazine peaks were consistently detected at a retention time of 5.7 min (Figure S10). Hydrazine oxidation selectivity (%) was calculated by the following equation:

$$Hydrazine Selectivity (\%)=\frac{Q_{reaction}}{Q_{total}}\times100$$

$Q_{total}$: Total charge passed during electrochemical experiments

$Q_{reaction}$: Charge passed toward hydrazine oxidation reaction

Regarding 4 electrons are involved (N_2_H_4_ + 4OH^−^ → N_2_ + 4H_2_O + 4e^−^, *E*^0^ = −0.33 V vs. RHE) in the reaction, $Q_{reaction}$ can be calculated by the following equation:

$${Q_{reaction}}=n_{N_{2}H_{4},consumed}\times4\times F$$

$F$: Faraday constant (96485 C mol^−1^)

Proton transport efficiency was calculated by subtracting the hydrazine selectivity from 100%.

**Theoretical limiting current density**

Theoretically, assuming complete occupation of hydrogen in the octahedral interstitial sites at the anolyte-facing side of Pd (PdH_1_) and full hydrogen depletion at the catholyte-facing side of Pd, the maximum hydrogen concentration in Pd can be estimated from the atomic concentration of Pd:

$$C_{Pd}= \frac{\rho_{Pd}}{M_{Pd}}=\frac{12.0 g {cm}^{-3}}{106.42 g {mol}^{-1}}\approx0.113 mol {cm}^{-3}$$

where $C_{Pd}$, $\rho_{Pd}$, and$M_{Pd}$ is the atomic concentration, density, and molecular weight of Pd, respectively. Under the idealized condition of a full hydrogen concentration gradient across the Pd foil (PdH_1_ → Pd), the hydrogen flux can be estimated using Fick’s law:

$$J_{H}= -D\frac{dC_{H}}{d_{x}}\approx D\frac{\Delta C_{H}}{L}$$

where *J_H_* is hydrogen flux, *D* is the hydrogen diffusivity in PdHₓ (1.3 × 10^−7^ cm^2^ s^−1^), *C_H_* is the atomic concentration of H and *L* is the foil thickness (0.01 cm). Using the theoretical hydrogen flux becomes:

$$J_{H}= 1.3 \times{10}^{-7}\times\frac{0.113}{0.01}\approx1.470 \times{10}^{-6} mol {cm}^{-2} s^{-1}$$

The corresponding proton-equivalent current density is given by:

$$j= FJ_{H}$$

where *j* is the current density, and *F* is the faradaic constant (96485 C mol^−1^). By substituting these values into the equation, we obtain:

$$j= 96485\times1.47\times{10}^{-6}\approx0.142 A {cm}^{-2}$$

thus, yielding a theoretical limiting current density of:

$$j_{max} \approx140 mA {cm}^{-2}$$

**D_2_O isotope experiment and quantification of water-derived protons by ^1^H NMR**

To evaluate electrochemically driven proton transport through the bipolar H-Pd membrane, the accumulation of water-derived protons in the D_2_O catholyte was monitored by ^1^H NMR. The experiment was performed using the bipolar H-Pd membrane cell configuration, in which the catholyte and anolyte compartments were separated by the Pd foil membrane. 0.1 M NaClO_4_ in 99.9% D_2_O (15 mL) was used as the catholyte, while H₂O was used as the anolyte. The catholyte was sampled under four conditions: before operation (Before), after 20 h at open-circuit voltage (OCV 20 h), and after electrolysis at −20 mA (−13.3 mA cm⁻²) for 2 h (20 mA 2 h) and 20 h (20 mA 20 h), respectively. The concentration of water-derived protons in the D_2_O catholyte was quantified by ^1^H NMR using DMSO as an internal standard. A 500 mM DMSO stock solution was prepared in D_2_O. For each NMR sample, 450 µL of the collected catholyte was mixed with 50 µL of the DMSO stock solution, giving a final DMSO concentration of 50 mM (Figure 3a). Calibration standards were prepared in D_2_O with fixed 50 mM DMSO and H_2_O concentrations of 0, 100, 250, 500, 750, and 1000 mM (Figure 3a). ^1^H NMR spectra were acquired without water suppression to preserve the H_2_O/HOD resonance. The singlet of DMSO at ~2.72 ppm was used as the internal standard and its integral was normalized to 100. The H_2_O/HOD resonance at ~4.75 ppm was integrated and used to construct the calibration curve.

**Li-NRR with HOR experiment**

The Li-NRR with HOR experiment setup was constructed with reference to the “continuous-flow reactor” reported by Fu et al.^39^ A custom-made poly ether ether ketone (PEEK) electrolyte flow channel was sandwiched between a cathode current collector and a titanium anode current collector within a commercial frame (Dioxide Materials, 5 cm^2^ MEA type-CO_2_ Electrolyzer) (Figure S11,12). Nitrogen (N_2_, 99.9999%, DIG AIRGAS) was fed to the cathode and hydrogen (H_2_, 99.999%, KSTECH) to the anode through N_2_ and H_2_ purifiers (NuPure), respectively, each at a flow rate of 15 sccm controlled by a mass flow controller (MFC, Bronkhorst). The system maintained a slight back pressure of approximately 10–12 mbar; the N_2_ outlet line was bubbled through an acid trap (30 mL of 0.05 M H_2_SO_4_ in a 50 mL graduated cylinder) to capture gas phase ammonia, while the H_2_ outlet was vented through an oil bubbler. The electrode assembly consisted of an Stainless steel cloth (SSC) cathode (SUS 316, 325 mesh), a Pt mesh anode, and a Pt wire pseudo-reference electrode. Prior to use, the SSC was cleaned by sequentially sonicating three times for 15 min each in DI water, EtOH, and acetone, followed by vacuum drying at room temperature overnight. The electrolyte flow channel provided an effective active area of 4 cm^2^. A peristaltic pump (ESM Tech) circulated the electrolyte between a three-neck round-bottom flask (3-neck RBF) and the reactor at a rate of 2 mL min^–1^ via PFA tubing. Prior to filling with electrolyte, the reactor was purged with N_2_ and H_2_ at 100 sccm for 30 min. Electrolytes were prepared inside an Ar-filled glovebox in which the O₂ concentration was maintained at ≤0.3 ppm (typically ~0.1 ppm) and H₂O at the detection limit (0 ppm). After preparation, the electrolyte was transferred to a 3-neck round-bottom flask (3-neck RBF), sealed with a rubber septum, removed from the glovebox, and connected to the reactor. Throughout the experiments, the 3-neck RBF was continuously purged with N₂ at 15 sccm. The electrolyte was stirred at 400 rpm in the 3-neck RBF. All electrochemical measurements were performed using a Bio-Logic VSP-3e.

**Li-NRR with bipolar H-Pd membrane experiment**

Li-NRR with a bipolar H-Pd membrane and aqueous anolyte experiments were conducted by inserting a Pd foil between the electrolyte flow channel and the anode to separate the catholyte and anolyte (Figure S13,14). Electrical contact to the Pd foil was taken externally using Cu tape. The active area of the Pd foil was 4 cm^2^. For the anode, a platinized titanium screen mesh (Pt/Ti mesh, Dioxide Materials) was coated with IrO_2_ to catalyze the OER. An IrO_2_ catalyst ink was prepared by mixing 30 mg of IrO_2_, 1 mL of IPA, and 300 μL of Nafion solution and was sprayed onto the Pt/Ti mesh to a loading of 1 mg cm^–2^ using a spray gun (Gunpiece GP-2, Fuso Seiki) to fabricate the anode. A nylon membrane filter was placed between the Pd foil and the anode to prevent direct electrical contact. The aqueous anolyte was circulated at 10 rpm using a peristaltic pump (ESM Tech), while all other procedures, including the non-aqueous catholyte and cell operation, followed those described for the Li-NRR with HOR experiments. Before each Li-NRR experiment, hydrogen was pre-loaded in the bipolar Pd membrane. The Pd foil and the anode were connected in a two-electrode configuration, and the total charge passed (23,376 C cm^–2^) was adjusted in proportion to the amount of Pd. Hydrogen absorption was performed by applying a constant current of –16.2 mA cm^–2^ for 1 h to a 0.025 mm Pd foil and –64.9 mA cm^–2^ for 1 h to a 0.1 mm Pd foil (Figure S15). Again, during Li-NRR, the bipolar H-Pd membrane was not connected to the electrode simply to act as a membrane.

**Cleaning procedures**

After the experiments, the reactor and the 3-neck RBF were thoroughly rinsed with DI water, boiled in DI water for 1 h, rinsed with EtOH, and then dried overnight in an oven at 60 °C. PFA tubing used for electrolyte circulation was flushed sequentially with tap water, DI water, EtOH, and acetone, and subsequently dried overnight at 60 °C. The Pt mesh was cleaned in piranha solution (H_2_SO_4_/H_2_O_2_), boiled in DI water, rinsed with EtOH, and dried overnight at 60 °C.

**Preparation of anhydrous solvents**

THF and EtOH were dried over activated molecular sieves. Before use, the molecular sieves were cleaned by sonicating them three times for 20 min each in EtOH and acetone, and then dried in a vacuum oven at 200 °C for 24 h. The activated sieves were subsequently transferred into THF and EtOH inside the glovebox and allowed to stand for at least 7 days before use.

**Phenoxide proton carrier experiments**

Phenoxide (PhO^–^) and PhOH were quantified by ^1^H NMR (Bruker Ascend Evo 500) using TMB as an internal standard (Figure S16). Calibration samples were prepared by mixing 50 µL of the electrolyte (1.0 M LiBF_4_ and 43 mM EtOH in THF) containing PhOH at 300, 200, 100, 50, 25, 10, or 0 mM with 50 µL of 50 mM TMB in DMSO-d_6_ and 400 µL of DMSO-d_6_. Spectra were acquired using the Bruker IconNMR automation software with a ^1^H NMR method (number of scans: 64). The singlet of TMB at ~6.1 ppm was integrated and normalized to 150, and the integral of the meta-H resonance of PhOH between 7.1 and 7.2 ppm was used to construct the calibration curve (Figure S16c). For phenoxide proton-carrier experiments, aliquots of the electrolyte were taken during operation for analysis while minimizing exposure to air and moisture, which would convert PhO^–^ to PhOH. HPLC vials (Agilent Technologies) with septum caps were pre-loaded into the glovebox, sealed, and taken out immediately before sampling. Electrolyte was withdrawn from the 3-neck RBF using a syringe and transferred into the HPLC vial, which was then promptly returned to the glovebox. Inside the glovebox, ^1^H NMR samples were prepared using anhydrous DMSO-d_6_, and the TMB internal standard solution was also prepared in anhydrous DMSO-d_6_.

**NH_3_ quantification**

NH_3_ was quantified using an appropriate method for each sample, namely the indophenol blue method, ^1^H NMR, or IC. In the phenoxide proton carrier experiments, ammonia was quantified by the indophenol blue method rather than ^1^H NMR, because the ^14^NH_4_^+^ resonance overlaps with the PhO^–^/PhOH signals in the ^1^H NMR spectra. The blank electrolyte consisted of 1 M LiBF_4_ in THF containing 100 mM PhOLi. For the colorimetric assay, 1.8 mL of DI water, 0.2 mL of electrolyte, 1.6 mL of 1 M NaOH(aq) containing 5 wt% salicylic acid and 5 wt% sodium citrate, 0.4 mL of 10-fold diluted NaClO, and 0.12 mL of 1 wt% sodium nitroferricyanide(III) dihydrate aqueous solution were added sequentially. The mixture was then incubated in the dark at room temperature for 2 h. After incubation, the solution was transferred to a quartz cuvette and analyzed by UV–vis spectroscopy (PerkinElmer Lambda 465). Spectra were recorded from 450 to 900 nm, and the absorbance at 655 nm was used for quantification. A calibration curve was constructed using ^14^NH_4_Cl solutions with concentrations of 9.38, 18.8, 37.5, 75.0, 150, and 300 µM (Figure S17). For all other experiments employing EtOH as the proton carrier, ammonia in the non-aqueous electrolyte was quantified by ^1^H NMR, whereas the ammonia captured in the acid trap was quantified by IC, because its concentration was too low to be reliably measured with our ^1^H NMR protocol. ^1^H NMR spectra were acquired on a Bruker Ascend NEO 500 using the Bruker IconNMR automation software with a water-suppression method (number of scans: 256; delay: 2 s; spectral width: 20.8216 ppm; transmitter frequency offset: 4.7 ppm). NMR samples were prepared by mixing DMSO-d_6_ (700 µL), deionized water (50 µL), 4 M H_2_SO_4_ in DMSO-d_6_ (50 µL), 30 mM MA in DMSO-d_6_ (125 µL), and electrolyte (125 µL), where MA served as the internal standard. Calibration curves were constructed using ^14^NH_4_Cl standards at 0, 3.75, 7.5, 15, 30, 60, and 120 mM. The integral of the MA singlet at 6.2–6.3 ppm was normalized to 100 for quantification (Figure S19). IC measurements were performed on a Metrohm Eco IC equipped with a cation-exchange column (Metrosep C6), following the procedure reported by the Ib Chorkendorff group^39^. To ensure chemical compatibility with THF-containing samples, all flow components and tubing were made of PTFE or PEEK. The eluent was 3.3 mM HNO_3_ containing 10 vol% acetonitrile, and was sonicated prior to use to degas. The method parameters were: eluent flow rate 0.9 mL min^–1^, sample injection volume 20 µL, and data acquisition time 25 min. Calibration was performed with ^14^NH_4_Cl standards at 0.05, 1, 5, 10, and 20 ppm to generate calibration curves (Figure S20). Acid-trap samples were mixed 1:1 with 100 mM LiOH prior to IC analysis.n

**Faradaic efficiency**

FE was calculated with the following equation:

$$FE=\frac{3\cdot F\cdot C\cdot V}{Q}$$

C: Concentration of ammonia

V: Volume of the electrolyte after the reaction

Q: Total charge passed during electrochemical experiments

**Surface characterization**

Surface was characterized by scanning electron microscopy (SEM) using a Thermo Fisher Scientific Apreo 2 S HiVac system. SEM/energy-dispersive X-ray spectroscopy (SEM/EDS) analysis was performed using an AZtecLive ULTIM^®^ Max system. X-ray diffraction (XRD) patterns were collected using a Bruker D2 Phaser diffractometer with Cu Kα radiation.

**Pd quantification**

The Pd content was quantified using inductively coupled plasma optical emission spectroscopy (ICP-OES, PerkinElmer Avio 220MAX). A calibration curve was constructed by diluting a commercial ICP standard solution to concentrations of 0.05, 0.1, 0.2, 0.5, 1, and 2 ppm (Figure S32). After the extended durability operation, the electrolyte sample was diluted 100-fold prior to ICP-OES analysis.

**Energy efficiency**

To evaluate the energy efficiency of our water-fed ammonia synthesis cell, we follow the thermodynamic approach introduced by Fu *et al.* for Li-mediated ammonia synthesis using hydrogen oxidation at the anode^39^. We first define the standard Gibbs free energy of reaction, $\Delta G_{R}^{0}$, and the corresponding reversible cell voltage, $V_{cell}^{0}$, for the key chemical reactions using tabulated standard Gibbs free energies of formation. The standard Gibbs free energy of water electrolysis is obtained from the formation free energy of liquid water:

$$H_{2}O_{\left( l \right)}\to H_{2_{\left( g \right)}}+0.5O_{2_{\left( g \right)}}$$

$$\Delta G_{R,\text{H}_{\text{2}}\text{ syn}}^{0}=\sum_{i} \nu_{i}\Delta G_{f,i}^{0}=-\Delta G_{f}^{0}\left( H_{2}O_{\left( l \right)} \right)$$

Here, $\nu_{i}$ and $\Delta G_{f,i}^{0}$ are the stoichiometric coefficients and the standard Gibbs free energies of formation of species i, respectively. Using literature values at 298 K, the reversible cell voltage for water splitting is

$$V_{\text{H}_{\text{2}}\text{ syn}}^{0}=\frac{\Delta G_{R,\text{H}_{\text{2}}\text{ syn}}^{0}}{zF}=\frac{237129}{2\cdot96485}=1.23 \text{V}$$

where $z=2$ is the number of electrons transferred and $F$ is the Faraday constant. Next, we consider ammonia synthesis from nitrogen and hydrogen:

$$0.5N_{2_{\left( g \right)}}+1.5H_{2_{\left( g \right)}}\to{NH}_{3_{\left( g \right)}}$$

$$\Delta G_{R,\text{N}\text{H}_{\text{3}}\text{ syn}}^{0}=\sum_{i} \nu_{i}\Delta G_{f,i}^{0}=\Delta G_{f}^{0}\left( {NH}_{3_{\left( g \right)}} \right)$$

which corresponds to the reversible voltage

$$V_{\text{N}\text{H}_{\text{3}}\text{ syn}}^{0}=\frac{\Delta G_{R,\text{N}\text{H}_{\text{3}}\text{ syn}}^{0}}{zF}=\frac{-16400}{3\cdot96485}\approx-0.06 \text{V}$$

using the tabulated value $\Delta G_{f}^{0}\left( {NH}_{3_{\left( g \right)}} \right)=-16.4 \text{kJ mo}\text{l}^{\text{-1}}$.^1^ Here $z=3$ electrons are required to form 1 mol of NH_3_. The overall reaction in which water is the ultimate proton source for ammonia synthesis:

$$0.5N_{2_{\left( g \right)}}+1.5H_{2}O_{\left( l \right)}\to{NH}_{3_{\left( g \right)}}+0.75O_{2_{\left( g \right)}}$$

The corresponding standard Gibbs free energy of reaction is

$$\Delta G_{R,\text{N}\text{H}_{\text{3}}\text{ to }\text{H}_{\text{2}}\text{O}}^{0}=\nu_{\text{N}\text{H}_{\text{3}}}\Delta G_{f}^{0}\left( {NH}_{3_{\left( g \right)}} \right)-\nu_{\text{H}_{\text{2}}\text{O}}\Delta G_{f}^{0}\left( H_{2}O_{\left( l \right)} \right)$$

$$\Delta G_{R,\text{N}\text{H}_{\text{3}}\text{ to }\text{H}_{\text{2}}\text{O}}^{0}=-16.45+1.5\times237.129=339.244 \text{kJ mo}\text{l}^{\text{-1}}$$

which leads to a thermodynamic cell voltage of

$$V_{\text{N}\text{H}_{\text{3}}\text{ to }\text{H}_{\text{2}}\text{O}}^{0}=\frac{\Delta G_{R,\text{N}\text{H}_{\text{3}}\text{ to }\text{H}_{\text{2}}\text{O}}^{0}}{zF}=\frac{339.244\times{10}^{3}}{3\cdot96485}=1.17 \text{V}$$

This voltage represents the minimum (reversible) electrical potential required to drive the global reaction when water provides the protons and air (or N_2_) supplies nitrogen, consistent with the analysis by Fu *et al.*^39^ We define the energy efficiency for ammonia synthesis as the ratio between the chemical energy stored in the produced NH_3_ and the total electrical energy consumed by the electrochemical cell:

$$EE_{\text{N}\text{H}_{\text{3}}}=\frac{E_{\text{out}}}{E_{\text{in}}}$$

Assuming that the desired product is NH_3_ and that O_2_ is a by-product without economic value, the ideal chemical energy output associated with $n_{\text{N}\text{H}_{\text{3}}}$ moles of ammonia is

$$E_{\text{out}}=n_{\text{N}\text{H}_{\text{3}}}\Delta G_{R,\text{N}\text{H}_{\text{3}}\text{ to }\text{H}_{\text{2}}\text{O}}^{0}=n_{\text{N}\text{H}_{\text{3}}}zFV_{\text{N}\text{H}_{\text{3}}\text{ to }\text{H}_{\text{2}}\text{O}}^{0}$$

The Faradaic efficiency to ammonia is defined as

$$FE_{\text{N}\text{H}_{\text{3}}}=\frac{3Fn_{\text{N}\text{H}_{\text{3}}}}{Q_{\text{tot}}}\quad\Rightarrow\quad n_{\text{N}\text{H}_{\text{3}}}=\frac{FE_{\text{N}\text{H}_{\text{3}}}Q_{\text{tot}}}{3F}$$

where $Q_{\text{tot}}$ is the total charge passed through the cell (absolute value).

$$E_{\text{out}}=FE_{\text{N}\text{H}_{\text{3}}}Q_{\text{tot}}V_{\text{N}\text{H}_{\text{3}}\text{ to }\text{H}_{\text{2}}\text{O}}^{0}$$

$$E_{\text{in}}=\int_{0}^{\tau} V_{\text{cell}}\left( t \right) I\left( t \right) dt$$

In our system, water oxidation at the anode and nitrogen reduction at the cathode occur in a single electrochemical reactor. Therefore, there is no separate H_2_ generation step, and the electrical energy input is simply the time integral of the cell voltage multiplied by the current:

$$\overline{V}_{\text{cell}}=\frac{1}{Q_{\text{tot}}}\int_{0}^{\tau} V_{\text{cell}}\left( t \right) I\left( t \right) dt$$

We define the average cell voltage during electrolysis as

$$E_{\text{in}}=Q_{\text{tot}}\overline{V}_{\text{cell}}$$

$$EE_{\text{N}\text{H}_{\text{3}}}=\frac{V_{\text{N}\text{H}_{\text{3}}\text{ to }\text{H}_{\text{2}}\text{O}}^{0}}{\overline{V}_{\text{cell}}} FE_{\text{N}\text{H}_{\text{3}}}$$

The energy efficiency of water-fed ammonia synthesis in our single-cell configuration:

$$EE_{\text{N}\text{H}_{\text{3}}}=\frac{1.17 \text{V}}{\overline{V}_{\text{cell}}} FE_{\text{N}\text{H}_{\text{3}}}$$

**Figure S1. Configuration of the electrochemical cells.** (**a**) H-type cell with the fixed inter-electrode distance for membrane performance testing. (**b**) Photograph of the two-electrode H-type cell used for the hydrogen absorption step. (**c**) Configuration of the electrochemical cell for decoupling membrane potential. (**d)** Photograph of the two-electrode setup to evaluate proton transport efficiency.

**Figure S2. CP for pre-hydrogen absorption.** Hydrogen was preloaded into Pd by applying a −100 mA current for 1 h.

**Figure S3. XRD patterns of H-Pd and Pd.** The H-Pd shows a similar peak with *β*-PdH, indicating hydrogen is incorporated into the Pd lattice.

**Table S1. Cell potentials using different types of membranes in various electrolytes.**

| Electrolyte | Membrane | Cell Potential (V) |
| --- | --- | --- |
| 0.1 M  HClO_4_ | w/o membrane | $-$1.97 |
|  | Nafion 211 | $-$1.99 |
|  | H-Pd | $-$2.09 |
|  | Pd | $-$3.53 |
|  | Pt | $-$3.90 |
|  | Ni | $-$4.00 |
| 0.1 M  Phosphate buffer | w/o membrane | $-$2.62 |
|  | Nafion 211 | $-$2.66 |
|  | H-Pd | $-$3.38 |
|  | Pd | $-$4.75 |
|  | Pt | $-$5.14 |
|  | Ni | $-$5.11 |
| 0.1 M  KOH | w/o membrane | $-$2.34 |
|  | Nafion 211 | $-$2.48 |
|  | H-Pd | $-$2.60 |
|  | Pd | $-$3.84 |
|  | Pt | $-$4.36 |
|  | Ni | $-$3.77 |

**Figure S4.** C**ell potentials measured using different types** **of membranes in neutral and alkaline electrolytes.** Cell potential profiles in (**a**) 0.1 M phosphate buffer and (**b**) 0.1 M KOH using various membranes.

**Figure S5. Potential measurements at different regions using H-Pd membrane.** Potential changes at (**a**) region 1, (**b**) region 3, (**c**) region 4, and (**d**) region 6. The potential was measured for 9 min. Potentials between the membrane (or electrode) and the reference electrode were measured under open-circuit conditions for the initial 2 min. From 2 to 7 min, the cell turned on. The cell was then switched off again from 7 to 9 min.

**Figure S6. Potential measurements at different regions using Pt foil.** Potential changes at (**a**) region 1, (**b**) region 3, (**c**) region 4, and (**d**) region 6. The potential was measured for 9 min. Potentials between the membrane (or electrode) and the reference electrode were measured under open-circuit conditions for the initial 2 min. From 2 to 7 min, the cell turned on. The cell was then switched off again from 7 to 9 min.

**Table S2.** **Potential distribution at each region in the electrochemical cell.**

|  | H-Pd  *E* (vs. RHE) | Pt  *E* (vs. RHE) |
| --- | --- | --- |
| Region 1 | −0.059 V | $-$0.048 V |
| Region 2 | 0.003 V | 0.003 V |
| Region 3 | 0.036 V | 1.865 V |
| Region 4 | −0.088 V | $-$0.029 V |
| Region 5 | 0.003 V | 0.003 V |
| Region 6 | 1.915 V | 1.926 V |
| Total *E* | 2.104 V | 3.874 V |

**Table S3. Ohmic resistances of catholytes and anolytes when H-Pd and Pt were used as the membrane.**

|  | Ohmic  resistance  (**Ω**) |
| --- | --- |
| H-Pd-Anolyte | 6.25 |
| H-Pd-Catholyte | 5.83 |
| Pt-Anolyte | 5.69 |
| Pt-Catholyte | 6.61 |

**Figure S7. Quantification of O_2_ gas.** (**a**) GC chromatogram of N_2_-balanced standard 100 ppm O_2_, 1000 ppm O_2_, and 10000 ppm O_2_ gases. GC chromatograms used to detect oxygen evolution at the catholyte-facing side of Pd, depending on (**b**) the hydrogen pre-loading into Pd and (**c**) current density.

**Figure S8.** Proton transport efficiency of H-Pd membrane at a current density of −100 mA cm^−2^.

**Figure S9.** H_2_ evolution selectivity obtained by GC analysis at the anolyte-facing side of Pd, depending on (a) the hydrogen pre-loading into Pd and (b) current density.

**Figure S10. Quantification of hydrazine.** (**a**) IC chromatograms of hydrazine standards (0.5–8 mM), catholyte before and after the reaction, and anolyte after the reaction. (**b**) A calibration curve for the quantification of hydrazine.

**Figure S11**. **Schematic illustration of the Li-NRR with HOR experiment and cell assembly.** (**a**) Schematic illustration of the experimental setup for the Li-NRR with HOR. N_2_ and H_2_ are supplied via MFCs to the SSC cathode and Pt mesh anode through their respective current collectors, while the non-aqueous electrolyte is circulated between them through the electrolyte flow channel using a peristaltic pump. The N₂ outlet stream is directed to an acid trap to capture gaseous NH_3_, and N_2_ is purged through the 3-neck RBF containing the electrolyte. (**b**) Exploded view of the electrochemical cell.

**Figure S12.** **Photograph of the experimental setup for the Li-NRR with HOR.**

**Figure S13.** **Schematic illustration of the Li-NRR with a bipolar H-Pd membrane and OER in anode compartment, and its cell assembly.** (**a**) Schematic illustration of the Li-NRR setup integrating a bipolar H-Pd membrane to separate the non-aqueous catholyte and the aqueous anolyte. Compared with the Li-NRR with HOR experiment, an aqueous anolyte is circulated through the anode current collector past an IrO_2_-coated platinized titanium mesh anode (labelled “IrO_2_ on Pt/Ti mesh”) using a peristaltic pump. (**b**) Exploded view of the electrochemical cell. The Pd foil membrane prevents crossover between the two electrolytes, while the nylon filter placed between the Pd foil and the anode blocks direct electrical contact.

**Figure S14.** **Photograph of experimental setup for the Li-NRR and OER, separated by a bipolar H-Pd membrane.**

**Figure S15.** **Potential profiles during pre-hydrogen absorption of the bipolar Pd membrane.** (**a**) Hydrogen charging potential profile for a 0.025 mm-thick Pd foil (active area 4 cm^2^) at −64.935 mA for 1 h. (**b)** Corresponding hydrogen charging potential profile for a 0.1 mm-thick Pd foil (active area 4 cm^2^) at −259.74 mA for 1 h.

**Figure S16.** **^1^H NMR calibration of PhOH using TMB as an internal standard.** (**a**) ^1^H NMR spectra of PhOH at different concentrations, recorded with TMB as the internal standard; spectra are normalized to the height of the TMB signal. (**b**) Expanded view highlighting the O–H and meta-H resonances PhOH. (**c**) Calibration curves constructed from the integrals of the PhOH O–H and meta-H peaks, obtained after normalizing the integral of the TMB singlet to 150. The concentrations of PhO^–^ and PhOH in the experiments were determined using the meta-H calibration curve.

**Figure S17.** **Calibration of the indophenol blue method for NH_3_ quantification.** The indophenol blue method was used exclusively to quantify NH_3_ in the catholyte during Li-NRR experiments employing the phenoxide proton carrier. (**a**) UV–vis spectra of indophenol blue solutions prepared with different NH_4_Cl concentrations. (**b**) Calibration curve constructed from the absorbance at 655 nm as a function of NH_4_Cl concentration. (**c**) Photographs showing the color of the indophenol blue solutions for each NH_4_Cl concentration after 2 h of incubation in the dark.

**Figure S18.** **Quantification of PhO^–^ and PhOH during phenoxide proton-carrier experiments.** Because THF evaporation and electrolyte withdrawal during intermediate sampling can slightly change the total solution volume, both the concentrations and absolute amounts of PhO^–^ and PhOH are compared. (**a**) Concentration ratios of [PhOH] to [PhO^–^]at each condition showing the evolution of [PhOH] only under Li-NRR to NH_3_ synthesis. (**b**) The amount of NH_3_ production at each stage. (**c**) ^1^H NMR spectra at each stage.

**Figure S19.** **^1^H NMR calibration of ^14^NH_4_^+^ using MA as an internal standard.** In all Li-NRR experiments employing EtOH as the proton carrier, ^1^H NMR was used to quantify NH_3_ in the non-aqueous electrolyte. (**a**) ^1^H NMR spectra obtained for solutions containing MA and ^14^NH_4_Cl at different concentrations. (**b**) Calibration curve constructed from the integral of the ^14^NH_4_^+^ triplet at 7.0–7.3 ppm, after normalizing the integral of the MA singlet at 6.2–6.3 ppm to 100.

**Figure S20.** **IC calibration for NH_3_ quantification.** NH_3_ captured in the acid trap was quantified by IC. (**a**) IC chromatograms obtained for NH_4_Cl standards at different concentrations. (**b**) Calibration curve constructed from the peak areas of the NH_4_^+^ peaks.

**Figure S21.** **Potential profiles of Li-NRR experiments with different configurations.** All experiments were carried out in a non-aqueous electrolyte of 1.0 M LiBF_4_ and 43 mM EtOH in THF under galvanostatic operation at −3 mA cm^−2^ for 2 h. (**a**) Li-NRR with a bipolar Pd membrane using a 0.025 mm Pd foil and a 0.1 M phosphate buffer anolyte. (**b**) Li-NRR with HOR.

**Figure S22.** **Li-NRR with a bipolar Pd membrane using a 0.1 mm Pd foil.** (**a**) Potential profile for Li-NRR operated at CP −3 mA cm^−2^ for 2 h using a 0.1 mm Pd foil, with 1 M LiBF_4_ and 43 mM EtOH in THF as the catholyte and 0.1 M phosphate buffer as the anolyte. (**b**) Comparison of NH_3_ FE using Pd foils with thicknesses of 0.025 mm and 0.1 mm.

**Figure S23.** **Photographs of SSC cathodes after Li-NRR with a bipolar H-Pd membrane.** All experiments used a 0.025 mm Pd foil and 0.1 M phosphate buffer as the anolyte. (**a**) SSC cathode after Li-NRR at CP −3 mA cm^−2^ for 2 h using 50 mL of catholyte (1 M LiBF_4_ and 43 mM EtOH in THF). (**b**) Same catholyte composition as in (**a**) after Li-NRR at −6 mA cm^−2^ for 2 h. (**c**) SSC cathode after Li-NRR using 1 M LiClO_4_ (instead of LiBF_4_). (**d**) SSC cathode after Li-NRR using 1 M LiTFSI (instead of LiBF_4_). (**e**) SSC cathode after Li-NRR using the same catholyte composition as in **a** with 100 mL catholyte under a potential cycling (−3 mA cm^−2^ for 1 min and 0 mA cm^−2^ for 1 min) for 12 h.

**Figure S24.** **Effect of anolyte pH on NH_3_ FE in Li-NRR with bipolar H-Pd membrane.** NH_3_ FE measured after 2 h of Li-NRR at CP −3 mA cm^−2^ using a 0.025 mm Pd foil and a catholyte of 1 M LiBF_4_ and 43 mM EtOH in THF. The anolyte was varied between 0.1 M HClO_4_, 0.1 M phosphate buffer, and 0.1 M KOH.

**Figure S25.** **Photographs of catholytes before and after Li-NRR with a bipolar H-Pd membrane.** All experiments were conducted using a 0.025 mm Pd foil and 0.1 M phosphate buffer as the anolyte. (**a**) Catholyte before electrolysis (1 M LiBF_4_ and 43 mM EtOH in THF). (**b**) Catholyte after Li-NRR at CP −3 mA cm^−2^ for 2 h using 50 mL of 1 M LiBF_4_ and 43 mM EtOH in THF. (**c**) Catholyte after Li-NRR at CP −6 mA cm^−2^ for 2 h with the same catholyte composition as in (**b**). (**d**) Catholyte after Li-NRR using 1.0 M LiClO_4_ (instead of LiBF_4_). (**e**) Catholyte after Li-NRR using 1.0 M LiTFSI (instead of LiBF_4_). In both (**d)** and (**e**), a distinct upper layer attributed to THF decomposition is visible. (**f**) Catholyte after potential cycling (−3 mA cm^−2^ for 1 min and 0 mA cm^−2^ for 1 min) for 12 h using the same catholyte composition as in **a** with 100 mL catholyte.

**Figure S26. 12 h cycling test.** The 12 h cycling test was performed using a catholyte of 1 M LiBF_4_ and 43 mM EtOH in THF (100 mL), a 0.025 mm Pd foil, and a 0.1 M phosphate buffer anolyte. (**a**) Potential cycling (−3 mA cm^−2^ for 1 min and 0 mA cm^−2^ for 1 min) applied for 12 h. (**b**) The amount of NH_3_ produced, quantified every 2 h. The reported NH_3_ values were calculated from the catholyte only (excluding the acid trap).

**Table S4.** **Water content measured by Karl Fischer titration and references.**

| Condition | Water (ppm) |
| --- | --- |
| Before | 52.9 ($\pm$ 4.4) |
| Open-circuit 3 h | 50.5 ($\pm$ 1.8) |
| Hydrogen charging 1 h  + Li-NRR 2 h | 53.5 ($\pm$ 1.1) |
| Hydrogen charging 1 h  + 12 h cycling test | 105.4 |
| 1 wt.% PMMA+Li-POM 0 h (Ref 1) | 53.7 |
| 1 wt.% PMMA+Li-POM 12 h (Ref 1) | 527.1 |
| AEM Initial (Ref 2) | ~150 |
| AEM 2 h (Ref 2) | ~6000 |
| CEM 2 h (Ref 2) | ~51255 |

The water content was measured for the electrolyte before electrolysis (“Before”), the electrolyte circulated under open-circuit conditions for 3 h without applying current (“Open-circuit 3 h”), the electrolyte after hydrogen charging for 1 h followed by Li-NRR for 2 h, and the electrolyte after hydrogen charging for 1 h followed by the 12 h cycling test. Measurements were performed in a dry room using a Karl Fischer coulometer (Metrohm 899 Coulometer). Electrolyte samples stored in an Ar-filled glovebox were taken out immediately before analysis and measured.

(Ref. 1) J. Miao, L. Wang, Y. Zhao, X. Chen, H. Li, Y. Shao-Horn, “Harnessing Lithium-Mediated Green Ammonia Synthesis with Water Electrolysis Boosted by Membrane Electrolyzer with Polyoxometalate Proton Shuttles,” Angewandte Chemie International Edition 64 (2025): e202503465, https://doi.org/10.1002/anie.202503465.

(Ref. 2) J. H. Kim, J.-E. Cha, H. Ju, Y.-W. Choi, J. Baek, J. G. Albers, J. Shim, S. H. Kim, K. Lee, H. C. Yoon, “Utilizing Water as a Proton Source for Sustainable Li-Mediated Electrochemical Ammonia Synthesis,” Chemical Engineering Journal 497 (2024): 154644, https://doi.org/10.1016/j.cej.2024.154644.

**Figure S27.** **Extended durability operation.** (**a**) Potential profiles of the working electrode and counter electrode during extended durability operation using 100 mL of DG-based catholyte containing 1 M LiBF_4_ and 43 mM EtOH, 0.1 M phosphate buffer anolyte, and a 100 μm-thick Pd foil membrane. The cell was operated under controlled cycling, consisting of 1 min of chronopotentiometry at −3 mA cm^−2^ followed by resting at 0 mA cm^−2^ until the cathode potential reached −2.3 V vs Pt wire. (**b**) Accumulated charge and NH_3_ FE during the extended durability operation. The NH_3_ FE was calculated from the total amount of NH_3_ quantified in the catholyte and acid trap.

**Figure S28. ^1^H NMR spectra of the DG-based catholyte before and after extended durability operation.** ^1^H NMR spectra of the DG-based catholyte recorded in acetone-d_6_ before and after extended durability operation. After operation, additional resonances appeared near 3.2 ppm, indicating the formation of DG-derived decomposition and/or oligomeric species. These spectral changes are consistent with the observed catholyte gelation after prolonged operation.

**Figure S29.** **XRD pattern of the Pd membrane after extended durability operation.** XRD pattern collected from the non-aqueous catholyte-facing side of the Pd membrane after extended durability operation. The dominant diffraction peaks are assigned to PdH_x_, whereas the weaker peaks correspond to metallic Pd. The result indicates that the hydrogenated PdH_x_ phase remained as the dominant crystalline phase after prolonged operation.

**Figure S30.** **SEM images of the 100 μm-thick Pd foil before and after extended durability operation.** SEM images of (**a**) bare Pd foil before electrochemical experiment, (**b**) Pd foil after pre-hydrogen absorption, (**c–e**) representative regions on the catholyte-facing side of the Pd foil after extended durability operation, and (**f**) the anolyte-facing side of the Pd foil after extended durability operation. Region-dependent surface morphologies, including localized deposits and roughened regions, were observed on the catholyte-facing side after prolonged operation, whereas the anolyte-facing side retained a relatively smooth surface morphology.

**Figure S31.** **SEM****–EDS analysis of the catholyte-facing side of the Pd foil after extended durability operation.** SEM images, elemental overlay maps, Pd, C, and O elemental maps, and corresponding EDS spectra collected from two representative regions, (**a**) and (**b**), on the catholyte-facing side of the 100 μm-thick Pd foil after extended durability operation.

**Figure S32.** ICP–OES calibration curve for Pd quantification.

**Figure S33.** **Comparison of energy efficiency with literature reports.** Energy efficiency is compared with selected Li-NRR studies that employ an aqueous anolyte, where protons are supplied via the OER at the anode.

(Ref. 1) H. Bemana, H. Schumann, M. McKee, S. Nozinovic, J. Daniels, R. Weisbarth, N. Kornienko, “Accelerating Lithium-Mediated Nitrogen Reduction Through an Integrated Palladium Membrane Hydrogenation Reactor,” Nature Communications 16 (2025): 6696, https://doi.org/10.1038/s41467-025-62088-z.

(Ref. 2) J. Miao, L. Wang, Y. Zhao, X. Chen, H. Li, Y. Shao-Horn, “Harnessing Lithium-Mediated Green Ammonia Synthesis with Water Electrolysis Boosted by Membrane Electrolyzer with Polyoxometalate Proton Shuttles,” Angewandte Chemie International Edition 64 (2025): e202503465, https://doi.org/10.1002/anie.202503465.

(Ref. 3) J. H. Kim, J.-E. Cha, H. Ju, Y.-W. Choi, J. Baek, J. G. Albers, J. Shim, S. H. Kim, K. Lee, H. C. Yoon, “Utilizing Water as a Proton Source for Sustainable Li-Mediated Electrochemical Ammonia Synthesis,” Chemical Engineering Journal 497 (2024): 154644, https://doi.org/10.1016/j.cej.2024.154644.

(Ref. 4) N. Lazouski, M. Chung, K. Williams, M. L. Gala, K. Manthiram, “Non-Aqueous Gas Diffusion Electrodes for Rapid Ammonia Synthesis from Nitrogen and Water-Splitting-Derived Hydrogen,” Nature Catalysis 3 (2020): 463–469, https://doi.org/10.1038/s41929-020-0455-8.

(Ref. 5) Ye, B., C. Burdis, V. Mints, Y. Zhou, A. Khobnya, G. Chen, R. Tort, J. Rietbrock, A. Kafizas, M. Ryan, M. Titirici, I. E. L. Stephens, “Continuous Ammonia Electrosynthesis from Nitrogen and Water in a Monolithic Pd Membrane-Based Flow Cell,” ACS Energy Lett. 11 (2026): 1907–1915, https://doi.org/10.1021/acsenergylett.5c03617.
